# Association of Genetic Variants With Migraine Subclassified by Clinical Symptoms in Adult Females

**DOI:** 10.3389/fneur.2020.617472

**Published:** 2021-02-12

**Authors:** Joe Kossowsky, Megan S. Schuler, Franco Giulianini, Charles B. Berde, Ben Reis, Paul M Ridker, Julie E. Buring, Tobias Kurth, Daniel I. Chasman

**Affiliations:** ^1^Department of Anesthesiology, Critical Care and Pain Medicine, Boston Children's Hospital, Boston, MA, United States; ^2^Division of Clinical Psychology and Psychotherapy, University of Basel, Basel, Switzerland; ^3^Harvard Medical School, Boston, MA, United States; ^4^RAND Corporation, Boston, MA, United States; ^5^Division of Preventive Medicine, Brigham and Women's Hospital, Boston, MA, United States; ^6^Computational Health Informatics Program, Boston Children's Hospital, Boston, MA, United States; ^7^Institute of Public Health, Charité–Universitätmedizin Berlin, Berlin, Germany

**Keywords:** latent class analyses, migraine diagnostic criteria, migraine with and without aura, migraine pain, genetic association analysis

## Abstract

Migraine is heritable and formally diagnosed by structured criteria that require presence of some but not all possible migraine symptoms which include aura, several distinct manifestations of pain, nausea/vomiting, and sensitivity to light or sound. The most recent genome-wide genetic association study (GWAS) for migraine identified 38 loci. We investigated whether 46 single-nucleotide polymorphisms (SNPs), i.e., genetic variants, at these loci may have especially pronounced, i.e., selective, association with migraine presenting with individual symptoms compared to absence of migraine. Selective genetic associations of SNPs were evaluated through a likelihood framework in the Women's Genome Health Study (WGHS), a population-based cohort of middle-aged women including 3,003 experiencing migraine and 18,108 not experiencing migraine, all with genetic information. SNPs at 12 loci displayed significant selective association for migraine subclassified by specific symptoms, among which six selective associations are novel. Symptoms showing selective association include aura, nausea/vomiting, photophobia, and phonophobia. The selective associations were consistent whether the women met all formal criteria for diagnostic for migraine or lacked one of the diagnostic criteria, formally termed probable migraine. Subsequently, we performed latent class analysis of migraine diagnostic symptoms among 69,861 women experiencing migraine from the WGHS recruitment sample to assess whether there were clusters of specific symptoms that might also have a genetic basis. However, no globally robust latent migraine substructures of diagnostic symptoms were observed nor were there selective genetic associations with specific combinations of symptoms revealed among weakly supported latent classes. The findings extend previously reported selective genetic associations with migraine diagnostic symptoms while supporting models for shared genetic susceptibility across all qualifying migraine at many loci.

## Introduction

In spite of heterogeneity in its presentation, migraine is highly heritable (1–3). The two predominant subclasses of migraine, migraine with typical aura (MA) and migraine without aura (MO) (4), appear to share some genetic influences with most loci from genome-wide association studies (GWAS) having been implicated in both subtypes (5–8). However, MA may have unique pathophysiology (9) and corresponding unique genetics (7, 10).

Beyond MA and MO, migraine may be further subclassified according to symptoms constituting migraine diagnosis (11) including pain character, photophobia, phonophobia, attack duration, and nausea/vomiting. Previously, we reported significant selective associations at 4 of 12 single-nucleotide polymorphisms (SNPs) from an early migraine GWAS (6) with migraine subclassified according to aura status or individual diagnostic symptoms (5). In principle, latent subclasses of aura status and the diagnostic symptoms may also underlie the heterogeneity of migraine presentation and may be accompanied by unique genetics (12–16). However, previous latent class analysis (LCA) of diagnostic symptoms among 6,265 twins (13) found that potential latent structure was consistent with a continuum of genetic liability rather than distinct genetics for each latent subclass.

Here, we expand on the existing literature (5, 10, 13), testing for selective associations with aura status and the diagnostic symptoms at 46 SNPs from 38 loci from the most recent GWAS (7). We also explored whether any selective genetic associations may be extended to self-reported migraineurs who do not meet full diagnostic criteria (11). Finally, we revisit migraine latent classes and potential corresponding selective genetics among the 46 SNPs in a sample with unprecedented power including 69,861 migraineurs.

## Materials and Methods

### Study Population

**Figure 1:**
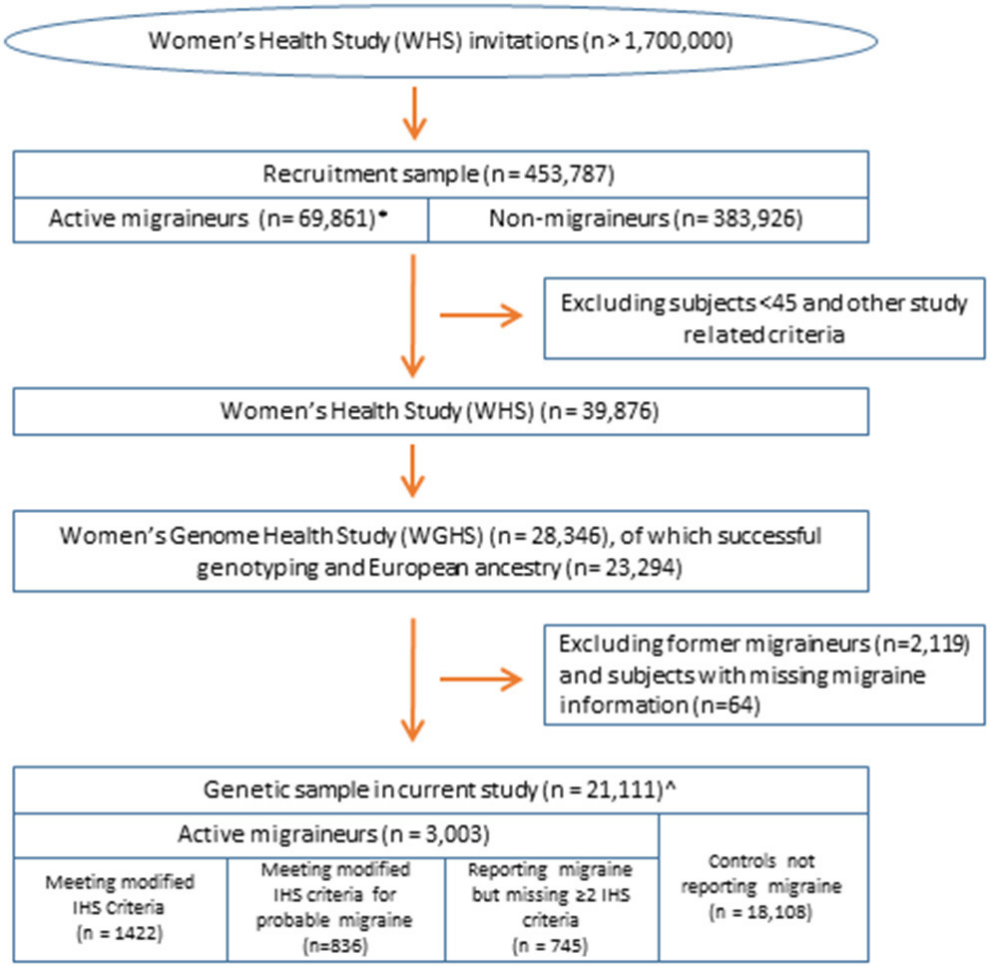
Overview of the study population. All samples are nested within the Women's Health Study (17). *Sample for latent class analysis. ^∧^Sample for analysis of selective genetic association by the likelihood procedure using the diagnostic symptoms or latent class assignments.

The current study leveraged data from the Women's Health Study (WHS) (Figure 1). The design, methods, and results of the WHS have been described in detail previously (18–20). In brief, the WHS was a randomized, placebo-controlled trial designed to test the benefits and risks of low-dose aspirin and vitamin E in the primary prevention of cardiovascular disease and cancer among 39,876 apparently healthy female healthcare professionals aged 45 or older at baseline. During 1992–1995, over 1.7 million female healthcare professionals were recruited to join the study, including women both younger and older than 45 (although the trial only included women older than 45), of whom 453,787 returned baseline questionnaires, which included questions for migraine assessment. The analytic sample for assessing migraine latent classes was the subset of 69,861 baseline respondents with self-identified European ancestry who reported having a migraine in the year preceding recruitment. The sample for the genetic analysis is derived from the Women's Genome Health Study (WGHS) (17), a subset of randomized WHS participants including 23,294 WHS participants with whole genome genotype data and verified European ancestry.

### Migraine Assessment

Migraine in the WHS was assessed at baseline by self-report as described previously (5, 21). Briefly, participants were asked: “Have you ever had migraine headaches?” and “In the past year, have you had migraine headaches?” Based on their responses, participants were classified as having no history of migraine, “active” migraine, i.e., migraine experienced within the past year, or “prior” migraine, i.e., migraine experienced previous to the past year. Participants reporting active migraine were additionally asked about further symptoms of their migraine attacks related to the International Classification of Headache Disorders (ICHD) criteria, such as aura status, nausea/vomiting, phonophobia, and photophobia, and included frequency of migraine attacks (21). Responses to these questions allowed classification using modified ICHD-2 criteria as described previously, including the formal diagnosis of probable migraine, defined in the ICHD as missing just one of the diagnostic criteria (Supplementary Table 1) (21). The migraine frequency variable was dichotomized as fewer than six attacks/year compared with 6 or more attacks/year. Of the 23,294 WGHS participants for genetics, analysis was restricted to the 3,003 with active migraine and 18,108 reporting no migraine, excluding 2,119 reporting prior but not active migraine and 64 with missing migraine status.

### Genotype Data

Whole-genome genotype data were collected in the WGHS as described (17). Statistical modeling was applied to the 46 candidate SNPs identified in a recent GWAS (7) that mapped to 38 distinct genomic loci (see Supplementary Table 2). Among these SNPs, rs6724624 and rs10166942 (at TRPM8) were in high LD (*r*^2^ = 1). SNPs at *FHL5* were in moderate LD (*r*^2^ = 0.25 for rs4839827–rs7775721 and *r*^2^ = 0.54 for rs67338227–rs7775721). SNPs rs12135062, rs10166942, rs11031122, rs11172113, and rs17857135 were genotyped directly. Genotype information for the remaining SNPs and missing genotype for the genotyped SNPs was derived from imputation using MaCH v.1.0.16 to the 1000 Genomes cosmopolitan reference panel (version 1, phase 3, March 2012) (22). All imputed SNP genotypes (as maximum likelihood dose) were of high quality, with minimum imputation *R*^2^ ≥ 0.87.

### Statistical Analyses

#### Likelihood Framework for Testing Selective Genetic Associations

A likelihood framework was used to evaluate the selectivity of associations between the GWAS SNPs and migraine subclassified according to individual diagnostic symptoms as described previously (5, 23). For each symptom, the WGHS sample was classified into three groups: (a) active migraineurs reporting the symptom, (b) active migraineurs not reporting the symptom, and (c) non-migraineurs, i.e., WGHS participants reporting having never experienced migraine. The Bayesian Information Criteria (BIC) was used to discriminate among six models for the association of each SNP with migraine compared with absence of migraine: (1) the “null” model of no association; (2) the “basic” model of association regardless of symptom-based subclass; (3) the “subset” model of association with migraine subtype defined by the presence and not absence a particular diagnostic symptom, (4) the “inverse subset” model of association with migraine subtype defined by the absence and not presence a particular diagnostic symptom, (5) the “general” model of a different magnitude association depending on presence or the absence of one of symptoms, and finally, (6) the “modifier” model assuming association with a given symptom conditional on being a migraineur. The significance of selected models was evaluated with a likelihood ratio test and a permutation method to address multiple testing as well as potential confounding. The permutation method provided corrected *p*-values for each SNP across all of the symptoms. These corrected *p*-values were further corrected for multiple testing across the 46 SNPs by the Šidák method. Subsequently, the magnitude of genetic associations with subclasses compared to non-migraineurs was assessed with logistic regression controlling for age and principal components of European ancestry substructure (see Supplementary Methods). All statistical analysis was performed in R (24).

#### Latent Class Analysis (LCA) of Symptoms From Migraine Diagnostic Criteria

Standard LCA was performed among active migraineurs (*N* = 69,861) and subsamples thereof derived from the WHS recruitment sample (*N* = 453,787) using the poLCA (25) package in R (24). Symptoms of the diagnostic criteria were encoded as binary variables (yes/no). PoLCA was iterated 50 times for each number of classes K ranging 2–15, using the BIC to determine an optimal model for each value of K (See Supplementary Methods). Genetic analysis was applied to the LCA results restricted to the subset of migraineurs and non-migraineurs in the WGHS subset. Selective SNP associations for a particular latent class (analogous to the “subset” model above) or for a particular LCA solution (analogous to the “general” model above) were evaluated by an extension of the likelihood framework for the diagnostic symptoms (See Supplementary Methods).

#### Other Statistical Procedures

Differences in demographics and health characteristics between migraineurs and controls were compared using ANOVA or Chi-square tests as appropriate.

### Data Use and Availability

All data collection and analysis were consistent with written informed consent in the WHS and approved by the Institutional Review Board (IRB) of Brigham and Women's Hospital. Public release of WGHS data is restricted by the IRB. However, access to data described in this work will be made available on a collaborative basis upon request.

## Results

Figure 1 shows the overall study design. We examined whether 46 genome-wide significant SNPs at 38 loci (Supplementary Table 2) identified in recent GWAS of migraine were selectively associated with migraine according to aura status and the individual diagnostic symptoms in three nested subsets of the Women's Genome Health Study (WGHS): fully qualifying migraineurs (*N* = 1,422), fully qualifying and probable migraineurs (*N* = 2,258), and all migraineurs (ICHD fully qualifying, ICHD probable migraineurs, and individuals reporting migraine but not meeting ICHD criteria) (*N* = 3,003) compared with 18,108 non-migraineurs. Demographic characteristics of the WGHS are shown in Table 1. The prevalence of aura and the diagnostic symptoms associated with migraine for the three nested samples WGHS migraineurs is shown in Table 2. Aura prevalence was roughly equivalent in all three subsets, whereas other symptoms, e.g., nausea/vomiting, were less prevalent at least partly owing to the lack of fulfillment of the diagnostic criteria.

**Table 1:** Demographic characteristics of the WGHS according to migraine status.

	**Full migraineurs^#^**	**Probable^#^ migraineurs**	**Other^#^ migraineurs**	**Controls^#^**	Effect size^*^	**P-val^†^**
***N***	**1,422**	**836**	**745**	**18,108**		
Age (yrs)	52.5 (5.4)	52.8 (5.9)	53.4 (6.2)	55.0 (7.3)	0.1	<0.001
BMI (kg/m^2^)	26.4 (5.3)	26.1 (4.9)	25.6 (4.6)	25.9 (4.9)	0.03	<0.001
Hormone replacement usage	693 (48.7%)	386 (46.2%)	355 (47.6%)	7,723 (42.7%)	0.02	<0.001
History of diabetes	29 (2%)	14 (1.7%)	12 (1.6%)	481 (2.7%)	0.02	0.06
History of hypertension	351 (24.7%)	176 (21.1%)	181 (24.3%)	4,465 (24.6%)	0.02	0.13
Ever smoker	663 (46.6%)	373 (44.6%)	339 (45.5%)	8,953 (49.4 %)	0.02	0.01
LDL cholesterol (mg/dl)	124.36 (34.3)	124.25 (32.9)	123.62 (33.4)	124.13 (34.2)	0.003	0.97
HDL cholesterol (mg/dl)	52.83 (14.9)	53.06 (14.4)	53.52 (14.7)	53.89 (15.1)	0.02	0.03
Triglycerides (mg/dl)	151.39 (89.9)	147.08 (92.9)	149.31 (98.3)	142.76 (91.7)	0.02	0.001

# *Mean (standard deviation) or N (%)*.

† *p-value from one-way ANOVA (continuous variables) or chi-square test (categorical variables)*.

* *Cohen's f: 0.1 denotes a small effect size, 0.3 denotes a medium effect size, and 0.5 denotes a large effect size*.

**Table 2:** Aura status and migraine characteristics of the three nested samples of the WGHS.

	**Full migraineurs (*N* = 1,422)**	**Full and probable migraineurs (*N* = 2,258)**	**All migraineurs (*N* = 3,003)**
**Aura or migraine symptom**	***N* (%)**	***N* (%)**	***N* (%)**
Aura	546 (38.4)	823 (36.4)	1,177 (39.2)
Pulsating pain	1,077 (75.7)	1,444 (63.9)	1,591 (53.0)
Unilateral pain	1,099 (77.3)	1,547 (68.5)	1,791 (59.6)
Phonophobia	867 (61.0)	1,161 (51.4)	1,232 (41.0)
Photophobia	1,162 (81.7)	1,671 (74.0)	1,976 (66.8)
Duration of 4–72 h	1,422 (100)	2,125 (94.1)	2,348 (78.1)
Nausea & vomiting	1,293 (90.1)	1,809 (80.1)	1,958 (65.2)
Pain aggravation by physical activity	797 (56.0)	970 (43.0)	1,017 (33.9)
Inhibition of daily activities	1,091 (76.7)	1,407 (62.3)	1,499 (49.9)
Migraine attack frequency ≥ 6/year	585 (41.1)	875 (38.8)	1,050 (35.0)

### Selectivity of SNP Associations for Aura and Other Migraine Characteristics

The results of the Bayesian Information Criterion (BIC)-based assessment of selective association with migraine subclassified by aura and the diagnostic symptoms at the 46 SNPs are shown in Table 3. The corresponding likelihood ratio test (LRT) *p*-values adjusted for multiple testing (*p*_*cor*_) are provided in Supplementary Table 3 (Methods and Supplemental Methods). In the WGHS, after correcting for multiple testing, fifteen SNPs (including the two *TRPM8* SNPs in high LD) were either significant for migraine overall regardless of symptoms or significantly selective for at least aura or one of the diagnostic symptoms for migraine. Overall, six SNPs that were not available in the previous selectivity analysis (5) were selective here: rs12260159 (*HPSE2*), rs4910165 (*MRVI1*), rs561561 (*IGSF9B*), rs11031122 (*MPPED2*), rs1024905 (*Near FGF6*), and rs17857135 (*RNF213*). In particular, SNPs rs12260159 and rs1024905 were selective for migraine without aura while the SNP at rs11031122 was selective for migraine accompanied with aura in at least one of the nested sets of migraine defined by diagnostic stringency.

**Table 3:** Genetic models^#^ selected with the BIC penalty for the three nested migraineur samples.

**Locus**	**chr:pos**	**SNP**	**Aura or migraine symptom^∧^^%^**									
			**Aura**	**Pulse**	**Unipain**	**Sound**	**Light**	**Longdur**	**Nausea**	**Agphys**	**Inhibit**	**Freq**
PRDM16	1:3159033	rs10218452	-	-	-	-	sub	-	sub	-	sub	i.sub
			basic^*^	basic^*^	basic^*^	basic^*^	sub^*^	basic^*^	sub^*^	basic^*^	sub^*^	i.sub^*^
			-	-	-	sub	sub	-	sub^*^	sub	sub^*^	-
PRDM16	1:3186748	rs12135062	-	-	-	-	-	-	-	-	-	-
			-	-	-	-	-	-	-	-	sub	i.sub
			-	-	-	-	-	-	-	-	sub	-
near TSPAN2-NGF	1:115134562	rs2078371	basic^*^	basic^*^	sub^*^	basic^*^	basic^*^	basic^*^	basic^*^	basic^*^	basic^*^	basic^*^
			basic	basic	sub^*^	sub	basic	basic^*^	basic^*^	basic^*^	basic^*^	sub^*^
			basic^*^	basic^*^	sub^*^	sub^*^	basic^*^	basic^*^	basic^*^	basic^*^	basic^*^	sub^*^
TRPM8	2:233911933	rs6724624^+^	-	-	-	-	-	-	-	-	-	-
			basic	basic^*^	basic	basic^*^	basic^*^	basic^*^	sub^*^	basic^*^	basic^*^	basic^*^
			basic^*^	basic^*^	basic^*^	basic^*^	basic^*^	basic^*^	basic^*^	basic^*^	basic^*^	basic^*^
TRPM8	2:233916448	rs10166942^+^	-	-	-	-	-	-	-	-	-	-
			basic	basic	basic^*^	basic^*^	basic^*^	basic^*^	sub^*^	basic^*^	basic^*^	basic^*^
			basic^*^	basic^*^	basic^*^	basic^*^	basic^*^	basic^*^	basic^*^	basic^*^	basic^*^	basic^*^
Near TGFBR2	3:30439067	rs6791480	-	-	-	-	-	-	-	-	sub	-
			-	-	-	-	-	-	-	-	-	-
			-	-	-	-	-	-	-	-	-	-
FHL5-UFL1	6:96594271	rs67338227	-	-	-	-	-	-	-	sub	-	-
			-	-	-	-	-	-	-	-	-	-
			-	-	-	-	-	-	-	sub	-	-
FHL5-UFL1	6:96609103	rs7775721	-	-	-	-	-	-	-	sub	-	-
			basic	basic	basic	basic	basic	basic	basic	basic	basic	basic
			i.sub	-	sub	-	-	sub	sub	sub	-	-
HPSE2	10:98942980	rs12260159	i.sub	-	-	-	-	-	-	-	-	-
			-	-	-	-	-	-	-	-	-	-
			-	-	-	-	-	-	-	-	-	-
MRVI1	11:10652497	rs4910165	-	-	-	-	-	-	-	-	-	-
			-	-	-	-	-	-	sub	-	-	-
			-	-	-	-	-	-	-	-	-	-
IGSF9B	11:133959811	rs561561	-	-	-	-	-	-	-	-	-	-
			-	-	-	i.sub	-	-	sub	-	-	-
			-	-	-	-	-	-	-	-	-	-
MPPED2	11:30525891	rs11031122	-	-	-	-	-	-	-	-	-	-
			-	-	-	-	-	-	-	-	-	-
			sub	-	-	-	-	-	-	-	-	-
Near FGF6	12:4408974	rs1024905	-	-	-	-	-	-	-	-	-	-
			i.sub	-	-	-	-	-	-	-	-	-
			sub	-	-	-	-	-	-	-	-	-
LRP1	12:57133500	rs11172113	-	-	-	i.sub	-	-	-	-	-	-
			basic^*^	basic	basic	basic^*^	basic^*^	basic^*^	basic^*^	basic^*^	basic^*^	basic^*^
			i.sub	basic	basic	basic	basic	basic	basic	basic	basic^*^	basic^*^
RNF213	17:80288362	rs17857135	basic	basic	basic	basic	basic	basic	basic	basic	sub	basic
			basic	basic	basic	basic	basic	basic	sub^*^	basic^*^	basic^*^	basic^*^
			-	-	sub	sub	-	sub	sub	-	-	-

# *Models (see Methods): “-” = null, basic, “sub” = subset, “i.sub” = inverse subset*.

*All models had significant LLR test p-values (≤0.05) after correction by permutations for testing across all characteristics (see Methods)*.

“*” *indicates models with significant LLR test p-values (≤0.05) after additional correction for the number of SNPs using the Šidák correction (see Methods)*.

∧ *Migraine characteristics: aura, pulse (= pulsation), unipain (= unilateral pain), sound (= phonophobia), light (= photophobia), longdur (= duration of 4–72 h), nausea/vomiting, agphys (= aggravation by physical activity), inhibit (= severity inhibits daily activities), freq (= ≥6 attacks/year)*.

% *Shading denotes diagnostic evidence for migraine as migraineurs meeting full ICHD criteria for migraine (unshaded rows), ICHD criteria for either full or probable migraine (“lightly shaded rows), or ICHD criteria for full or probable migraine as well as other self-reported migraine (heavily shaded rows) (see Methods)*.

+ *These SNPs at TRPM8 are in high LD (r^2^ = 1, Methods)*.

Seven of 15 of the significant BIC models would not have been evident among fully qualifying migraineurs only, likely due to power. For example, the preferential association with nausea/vomiting in rs6724624 and rs10166942 (both *TRPM8*) was absent when limited to full migraineurs but significant in the combined full and probable migraineurs (*p*_*cor*_ < 0.001) and all migraineurs (*p*_*cor*_ < 0.001, Supplementary Table 3). Further, rs11031122 (*MPPED2*), which was the only SNP to be preferentially associated with the migraine with aura (“subset” model), was only significant in the sample of all migraineurs (*p*_*cor*_ = 0.015). Two SNPs, rs561561 (*IGSF9B*) and rs4910165 (*MRVI1*), were both found to be selective for migraine characterized by nausea/vomiting (*p*_*cor*_ = 0.010 and 0.006, respectively) in the combined full and probable migraineurs. Selectivity for migraine without aura was found for rs1024905 (near *FGF6*) in combined full and probable migraineurs (*p*_*cor*_ = 0.014) but consistent with the null among full migraineurs alone.

However, for other loci, significance decreased when augmenting the sample of fully qualifying migraineurs with probable or other migraineurs. For example, selectivity of the rs12260159 (*HPSE2*) was significantly preferential for migraine without aura (“inverse subset”) among the fully qualifying migraineurs (*p*_*cor*_ = 0.002) but not selected for association by the BIC in the larger samples including non-qualifying, self-reported migraineurs. A similar pattern was observed for rs6791480 (near *TGFBR2*) and migraine characterized by inhibited daily activity due to pain (*p*_*cor*_ = 0.013) among fully qualifying migraineurs but null in the augmented samples.

Meanwhile, other SNPs such as rs2078371 (near *TSPAN2*) the model identified with the BIC changed from non-selective (i.e., “basic”) in the limited group to being preferentially associated with migraine characterized by sensitivity to sound in the larger samples (both *p*_*cor*_=0.001). The same pattern was found for rs10218452 (*PRDM16*). The increased selectivity by augmenting the fully qualifying migraineur sample with probable migraineurs is counter to the inference that the selective associations are restricted to severe migraineurs.

Using logistic regression, we assessed the magnitudes of the significant associations at the 15 SNPs, especially those that were selective (Supplementary Table 4). The estimated effects (i.e., logistic regression beta-coefficients) of some SNPs were greater in the absence compared with presence of aura or a particular diagnostic symptom, again suggesting that selective effects do not necessarily reflect association with more severe migraine. For example, SNPs rs7775721 (gene *FHL5-UFL1*) among all migraineurs, rs12260159 (gene *HPSE2*) among fully qualifying migraineurs, rs1024905 (near *FGF6*) among full and probable migraineurs, and rs11172113 (*LRP1*) among all migraineurs are more strongly associated with absence of aura compared to its presence. Additional stronger associations are observed for absence compared with presence for phonophobia (rs561561 and rs11172113) and migraine frequency i.e., ≥6 attacks/year (rs10218452 and rs12135062). By contrast, stronger associations in the presence compared with absence of aura or the diagnostic symptoms were also observed, and included the aura-specific association at rs11031122 (*MPPED2*) that was also noted previously in the discovery GWAS (7).

### Selectivity of SNP Associations With Latent Classes

**Figure 2:**
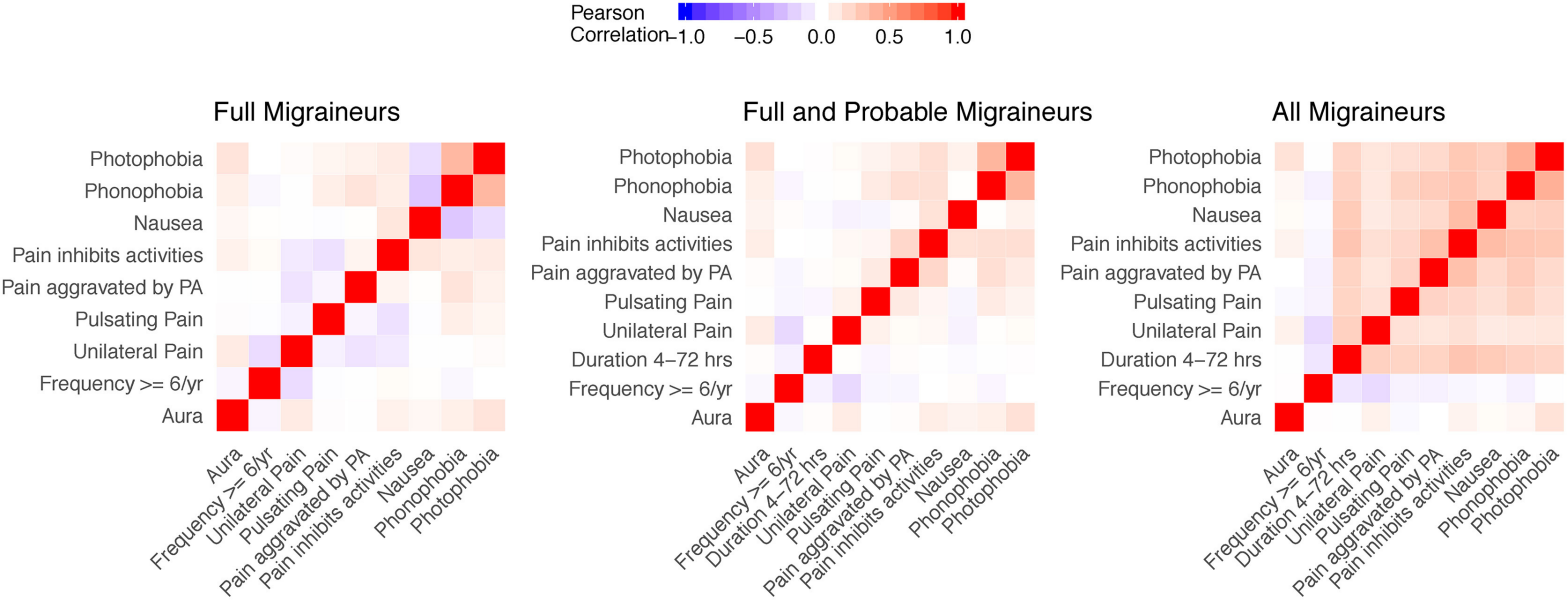
Correlation matrices of aura status and migraine diagnostic symptoms in the study sample. Correlations for aura and the diagnostic symptoms were evaluated in nested samples consisting of full migraineurs (*N* = 36,105), full and probable migraineurs (*N* = 54,011), and all migraineurs (*N* = 69,861). The feature of migraine duration lasting 4–72 h was removed from the full migraineur sample since this feature was present in all migraineurs as imposed by the diagnostic criteria. Unilateral pain, pulsating pain, pain aggravated by physical activity, and pain inhibiting daily activities relate to criterion C of the ICHD diagnostic criteria. Nausea/vomiting, photophobia, and phonophobia relate to criterion D of the ICHD diagnostic criteria.

Figure 2 shows the Pearson correlations of aura and other migraine characteristics in the three nested samples of the WHS recruitment population (N up to 69,861 migraineurs, Methods, Figure 1, Supplementary Table 5). Note that the diagnostic criteria will induce correlations in groups with full migraineurs and combined full and probable migraineurs, e.g., the strong correlations between photophobia and phonophobia.

**Figure 3:**
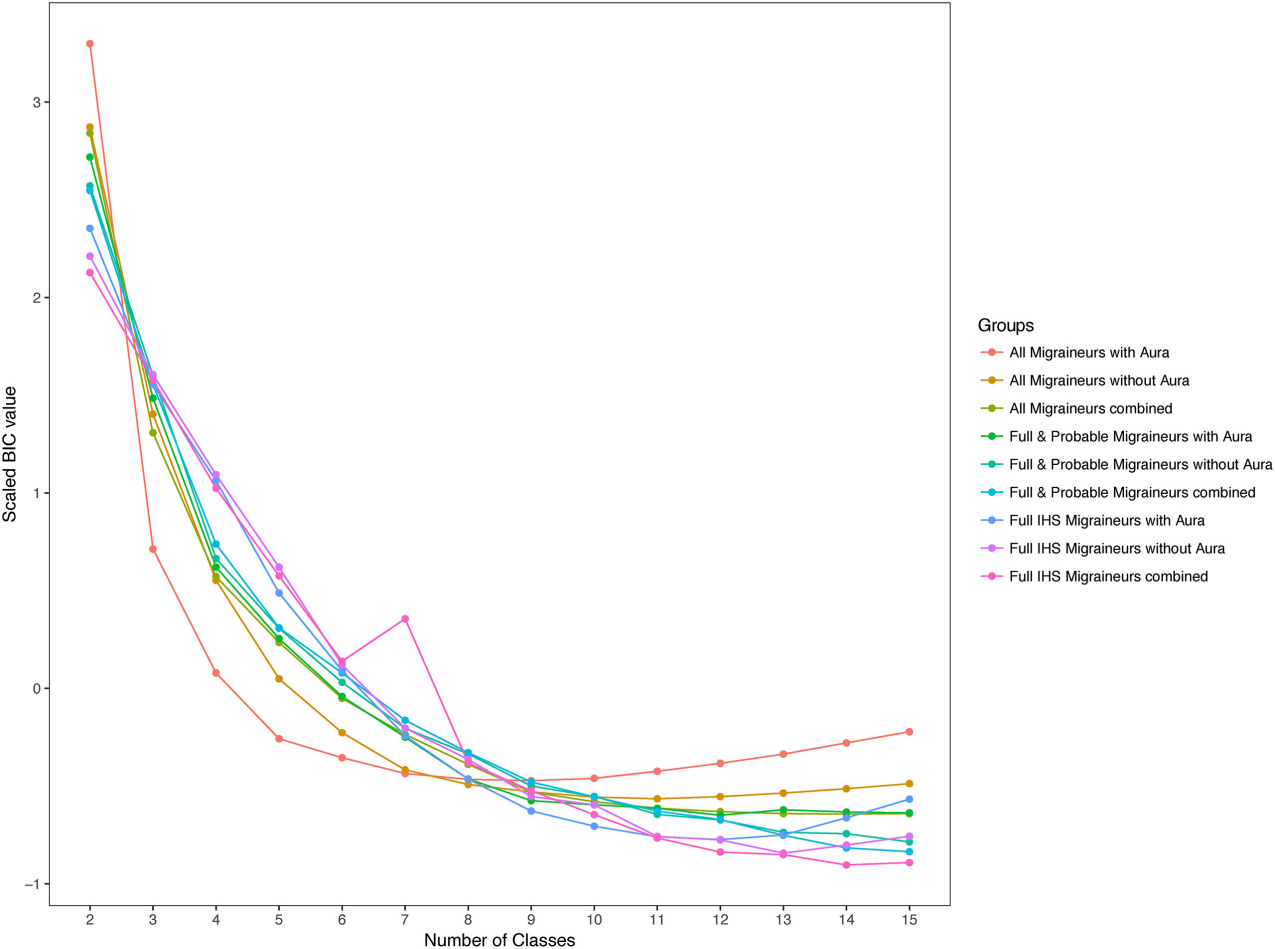
Scaled values of the Bayesian information criterion (BIC) for latent classes. BIC values for the 2-class to 15-class latent class solutions among migraineurs with and without aura in the three nested samples. BIC values were scaled across the nested samples to allow comparison.

Within these nested samples, we performed LCA using aura status and the diagnostic symptoms as binary manifest variables over total number of subclasses, K, ranging from 2 to 15. We also explored latent models in these samples further stratified by aura status. In each of these groups and for each value of K, LCA was performed with 50 random initializing frequencies. Yet none of the LCA models recurred for any K, indicating lack of truly stable solutions (26). Moreover, no clear support was found for an optimal value of K, as indicated by an absence of a clear optimum BIC value, except possibly among all WHS migraineurs reporting aura where a broad minimum was centered around K=8 (Figure 3). Including symptoms that are obligate for full ICHD migraine when applying LCA to the sample that included full, probable, and other active migraineurs may have precluded a robust solution. However, excluding these symptoms and repeating the entire LCA procedure with only aura status and the diagnostic symptoms for pain, nausea/vomiting, photophobia, and phonophobia did not reveal a clearly optimal K or a solution that recurred over the 50 iterations (not shown). Similarly, stratifying the LCA by age ≤45 v. >45, the former potentially relevant to greater prevalence of migraine among younger women did not reveal a reliable solution in the LCA (not shown).

In spite of the lack of a robust LCA solution, it remained possible that particular latent classes recur and moreover that such potential latent classes have pronounced, selective association with one or more of the candidate SNPs. However, having adapted the selective association hypothesis testing framework for latent classes (Materials and Methods), we found no selective associations meeting significance thresholds consistent with the large burden of multiple testing.

## Discussion

The current study focused on 46 GWAS migraine susceptibility SNPs (7), evaluating selective associations with migraine subclasses among adult women defined on the basis of individual symptoms of the diagnostic criteria (5, 10, 13, 14, 27). Six new selective associations were observed raising the total for this BIC-based model selection approach to 15 (5). The analysis further examined the influence of diagnostic stringency on selective associations. Meanwhile, in spite of exceptional power, LCA did not reveal robust overall substructure among diagnostic criteria of migraine, nor was there evidence of selective SNP associations with any individual latent classes that may have existed even in the absence of overall latent substructure.

Among six SNPs identified in the recent GWAS and chosen by the BIC model selection, three were selective for aura status (7). One of these SNPs, rs11031122, is the first reported specifically for MA and was noted for heterogeneity in comparing its association between MA and MO in meta-analyses that included data from the WGHS (7). Rs11031122 maps to the intron of the highly conserved gene for metallophosphoesterase-domain containing 2 (*MPPED2*) that is expressed in fetal brain (28), consistent with a role in neuronal development (29), although it is expressed also in adult non-brain tissues including aorta (30). The second SNP, rs1024905, was selective for migraine without aura in the combined full and probable migraineurs group. It maps near the *FGF6* gene that has been shown to play an important role in the regulation of cell proliferation, cell differentiation, angiogenesis, and myogenesis and is required for normal muscle regeneration (7, 31). The final new selective SNP pertaining to aura, rs12260159, which is in the intron of the heparanase 2 gene (*HPSE2*), was also selective for lack of aura but only in the stringently qualifying migraineur group. Heparinases are involved in tissue remodeling, and *HPSE2*, in particular, is expressed widely in fetal and adult central nervous system of mouse (32). Mutations in *HPSE2* have been associated with urofacial syndrome in humans, suggesting a potential developmental role (33).

The comparison of models selected across nested subsets of migraineurs with decreasing diagnostic stringency begins to address relationships among selectivity, genetic heterogeneity, and power. Among the most strongly associated SNPs, augmenting the sample of full migraineurs with probable migraineurs almost always resulted in greater selectivity and significance, most simply explained by a greater increase in power and minimal deterioration by potential heterogeneity. This interpretation is consistent with previous conclusions that probable migraineurs are genetically similar to full migraineurs (13). However, adding the “other” self-reported migraineurs who did not meet criteria for either full or probable migraine did not uniformly improve significance. These individuals may have aged out of some symptoms leading to misclassification of their status, they may have genetic background that is less susceptible to migraine at the candidate SNPs, or their self-reported migraine condition does not arise from genetic influences shared with migraine meeting ICHD criteria.

We have previously argued that selective genetic associations are not simply explained by associations with more severe migraine (5). If so, and were severity assessed by the presence of one or more specific symptoms, then the selective associations would have highlighted the same set of symptoms for each selective SNP. However, this was not the case. Nor were two measures of severity, the specific symptoms of high frequency migraine and/or aura, uniformly highlighted. At the same time, selective associations denoted by “inverse-subset” imply a stronger association in the absence rather than the presence of particular diagnostic symptoms and are possibly consistent with less severe migraine. Finally, in the current analysis, selectivity typically improved by augmenting the fully qualifying migraineurs with probable migraineurs, who may be viewed as experiencing less severe form of migraine. Notwithstanding the preceding argument, it is important to note that approximately half, i.e., only part, of the variation in the concordance rate for common migraine is attributable to genetic factors, while the remainder depends on environmental factors (1–3).

With up to 69,861 female migraineurs, our sample for LCA was over ten times larger than the sample in the previous analysis (13) and therefore better powered to detect much more subtle clustering. That previous study did not identify discrete latent structures but found evidence for a continuum of increasing numbers of symptoms, greater prevalence of aura, and male sex that was correlated with overall genetic liability. Although migraine in our study was coded slightly differently such that we could not evaluate symptom prevalence in the absence of self-reported migraine, our analysis also largely failed to reveal robust substructure, except possibly among migraineurs with aura who met stringent diagnostic criteria. Even in this group, there was no single model that recurred in the 50 randomly initialized iterations of the modeling procedure, suggesting that latent substructure among migraineurs remains elusive, if it exists at all.

While the main strengths of our study are the unprecedented sample size for the LCA and the large sample size for the genetic analysis for the diagnostic symptoms, the main limitation of our study is the self-reported ascertainment of migraine, which may result in misclassification. However, the demographic of our study population, female healthcare professionals, is known to provide accurate clinical information by self-reported questionnaire (34) and a previous study in the WHS showed good agreement between self-reported MO and classification of MO based on ICHD-2 criteria (21). Moreover, migraine status in the WGHS was used to identify the first consistent migraine susceptibility loci by GWAS (16) and to discover robust liability to stroke associated with MA (35). Further, our model selection was based on the BIC, which enforces a high stringency, and we used a permutation procedure to establish significance thresholds consistent with multiple hypothesis testing. Additional limitations pertain to the migraine ascertainment. Aura in the questionnaire was not distinguished from other prodromal phenomena, possibly leading to misclassification, and is at the higher limit of prevalence compared to other population-based surveys (36). Nor did the ascertainment provide longitudinal information about the symptoms. Again, the consequence is potential misclassification since it is impossible to distinguish whether symptoms may never have existed compared to whether they disappeared or possibly altered with age in our participants who were at least 45 years old (37–39). However, all of these possibilities would likely attenuate the selective associations we observe, and they may underlie the loss of some selective associations, e.g., for rs17857135 at gene *RNF213*, when augmenting the sample of full and probable migraineurs with other self-reported migraineurs in the selectivity analysis.

The diversity of symptoms qualifying for diagnosis of common migraine raises the possibility that the phenotypic heterogeneity is accompanied by underlying genetic heterogeneity (40). The findings here together with previous findings from ourselves and others support the notion that at least some of the heterogeneity in common migraine is influenced by genetics (5, 13). As more loci are discovered in future GWAS, we anticipate improving the understanding of both migraine pathophysiology and diagnosis through additional study of selective genetic associations.

## Data Availability Statement

The data analyzed in this study is subject to the following licenses/restrictions: The datasets used in this study, namely The Women's Genome Health Study and its parent cohort The Women's Health Study, are restricted from public access by the local IRB. However, the datasets are available through collaboration and no reasonable collaborative requests have been refused. Requests to access these datasets should be directed to dchasman@research.bwh.harvard.edu.

## Author Contributions

JK, FG, and DC: performed analysis. JK and DC: wrote manuscript. JK, MS, FG, CB, PR, JB, TK, and DC: critical revisions to the manuscript. MS, BR, CB, TK, PR, JB, and DC: supervision and advice. DC: secured funding. All authors contributed to the article and approved the submitted version.

## Conflict of Interest

TK has received honoraria from Lilly for providing methodological consultation and from Novartis for presenting a lecture in Neuroepidemiology. He further has received an honorarium from The BMJ for editorial services. DC has received an honorarium from Amgen for a presentation on migraine genetics. The remaining authors declare that the research was conducted in the absence of any commercial or financial relationships that could be construed as a potential conflict of interest.

## Abbreviations

GWAS: genome-wide association study
ICHD: International Classification of Headache Disorders
LCA: latent class analysis
LD: linkage disequilibrium
LRT: likelihood ratio test
MA: migraine with typical aura
MO: migraine without aura
SNP: single-nucleotide polymorphism.
