# Supplementary material for: Association of Genetic Variants With Migraine Subclassified by Clinical Symptoms in Adult Females
Source: Front Neurol. 2021 Feb 12;11:617472. doi: 10.3389/fneur.2020.617472 (PMC7907521; doi:10.3389/fneur.2020.617472)
Supplement: Supplementary file 1 [file Data_Sheet_1.PDF]

Supplemental Material: Kossowsky et al., 2020

Detailed statistical methodology

Likelihood framework for testing selective genetic associations

As described previously, we used a likelihood framework to discriminate among six potential inheritance models for the association of each GWAS SNP with migraine sub-classified according to the ICHD diagnostic criteria (1, 2). For each symptom, the WGHS sample was classified into three groups: a) active migraineurs reporting the symptom, b) active migraineurs not reporting the symptom, and c) non-migraineurs, i.e. WGHS participants reporting never experiencing migraine. The likelihoods of six different inheritance models, each specified by SNP minor allele frequencies in the three groups, were considered. The six models were: 1) the "null" model (1 degree of freedom [df]) assuming the same allele frequency in all three groups, i.e. no association; 2) the "basic" model (2df) assuming association with migraine *overall* regardless of subclass (i.e. a certain allele frequency among migraineurs and a different allele frequency among non- migraineurs); 3) the ''subset'' model (2df) assuming association of migraine subtype defined by the *presence* a particular diagnostic symptom (i.e. a certain allele frequency among migraineurs with a particular symptom and a different but identical allele frequency among both migraineurs without the symptom and non-migraineurs); 4) the ''inverse subset'' model assuming an association with migraine subtype defined by the *absence* of a particular symptom (i.e., a certain allele frequency among migraineurs lacking a particular symptom and a different but identical allele frequency among both migraineurs with the symptom and non-migraineurs); 5) the ''general'' model assuming *differential association* with migraine subtypes defined by the presence and the absence of one of symptoms (i.e., different minor allele frequencies in all three groups); and finally, 6) the ''modifier'' model assuming association with a given symptom conditional on having migraine (i.e., different minor allele frequencies in migraineurs with and without a given symptom and the weighted average of the two frequency estimates among the non-migraineurs).

For each SNP, the Bayesian information criterion (BIC) was used to select a best-fitting inheritance model in the WGHS data (3). The significance of a selected, non-null inheritance model was evaluated with the standard log-likelihood ratio (LLR) test statistic comparing the likelihood of a selected model with the likelihood of the null model. A permutation, step-down approach was used to account for multiple testing (6 models, 46 SNPs) in assessing the significance of the LLR test while accounting for correlation structure across all hypotheses as follows (4). This procedure began by deriving the null distributions of the LLR test p-values across all symptoms by repeating the model selection procedure for 10,000 random permutations of the genotype with respect to migraine status. Next, for each SNP, an empirical p-value was determined for selective association with each symptom as the proportion of LLR test p-values from the permutations that were smaller than or equal to the observed LLR-test p-value. These empirical p-values were used to order the corresponding symptoms from most to least significant selective association. Then, a step-down procedure was used to derive a p-value that was corrected across symptoms (still for each SNP at a time). The analytic p-value for the symptom with the most significant empirical p-value was compared to the distribution of the most significant analytic p-value across all symptoms from the permutations; and the corrected p-value for the symptom was computed as the proportion of these minimum permutation p-values that were less than the symptom's analytic p-value. If this empirical p-value was significant, then that symptom was excluded and the process was repeated for the symptom with the second most significant empirical p-value (4). Although each SNP had previously been identified on the basis of

genome-wide significant association with migraine in the large GWAS (5), i.e. $p < 5x10^{-8}$, these empirically corrected p-values were further corrected for testing across all 46 SNPs by the Šidák method. The strength of the migraine associations in the presence or absence of aura or the diagnostic symptoms was quantified by logistic regression adjusted for 10 eigenvectors of European ancestry population sub-stratification.

To explore the strength of evidence for migraine diagnosis on (potentially selective) genetic association with migraine, we performed the likelihood-based model selection on three nested groups of active migraineurs (see study design figure). The first group, termed "full migraineurs", met all diagnostic criteria for migraine by self-report according to modified ICHD-2 criteria (6); the second group, termed "full and probable migraineurs" consisted of the first group as well as migraineurs met all criteria but one (i.e. probable migraineurs); and the third group, termed "all migraineurs" consisted of the two former groups and other women reporting having active migraine but who did not meet at least two diagnostic criteria for full migraine, and included women reporting migraine aura but not headache.

Latent class analysis (LCA) of symptoms from migraine diagnostic criteria

Standard LCA was performed among active migraineurs in the WHS recruitment sample (n=69,861) and subsamples thereof using the poLCA (7) package in R. Symptoms of the diagnostic criteria were encoded as binary variables (yes/no). LCA requires pre-specification of the number of classes, K, which was varied from 2 to 15. For each value of K, poLCA was run 50 times with different random initialization of the symptom frequencies to ensure convergence and reduce the influence of local maxima. To determine the optimal number of classes K, we compared BIC values for models with minimum BIC across the 50 iterations for each value of K ranging from 2 to 15 classes (3, 8).

Selective genetic associations with latent classes

To assess potential selective genetic associations for latent classes, we borrowed from the likelihood approach described above for individual migraine symptoms, implementing likelihood ratio tests (LRTs) and the BIC to compare various inheritance models specified by the allele frequencies within the latent classes of migraineurs and the non-migraineurs. These tests of genetic association were performed among WHS migraineurs from the recruitment sample included in the LCA who were also included in the nested WGHS sub-sample combined with non-migraineurs from the WGHS (see study design figure). Three inheritance models, analogous to the "basic", "subset" and "general" models above, were evaluated for each class K. We refer to these models using slightly different terminology replacing the term "subset" with the term "sub-class" in the LCA setting. As above, the "basic" model corresponds to a significant association with migraine regardless of potential latent subclass. In testing the sub-class model, the allele frequency of a SNP in each sub-class of the K-class model, one at a time, was compared to the allele frequency among all other K-1 sub-classes and the non-migraineurs combined. These models had 2 degrees of freedom (2df) for each sub-class. The general model was specified by a different allele frequency in each of the K latent sub-classes of migraineurs for a K-class model and the non-migraineurs for K+1 degrees of freedom. All association models were compared to the null model of a uniform allele frequency among all migraineurs and non-migraineurs (1df). Thus, BIC model selection evaluated the "null", "sub-class", or "general" modes of inheritance. For hypothesis testing of selective associations, we prioritized the "maximum statistic test". In this test, for an optimal LCA model with K classes, we considered only the

sub-class with the most significant of the K (which would have been identified in BIC selected "sub-class" models), 1 df LRT (compared to the null model), and adjusted this p-value for multiple testing accounting for K tests using the Šidák method. For hypothesis testing with the general model, termed the "omnibus" test, we considered the K df LRT (compared to the null model). To implement these tests, allele frequencies within each latent class were calculated two ways, although the results were essentially the same for both. In the first, allele frequencies were estimated using by weighting the allele dose for each individual by the posterior probability of assignment to each sub-class in the latent class model. In the second, allele frequencies were estimated by assigning the entire allele dose to the maximum likelihood sub-class for each individual.

References

[1] Chasman DI, Anttila V, Buring JE, Ridker PM, Schurks M, Kurth T, International Headache Genetics C. Selectivity in genetic association with sub-classified migraine in women. PLoS genetics. 2014;10(2014):e1004366.

[2] Lee PH, Bergen SE, Perlis RH, Sullivan PF, Sklar P, Smoller JW, Purcell SM. Modifiers and subtype-specific analyses in whole-genome association studies: a likelihood framework. Hum Hered. 2011;72(2011):10-20.

[3] Schwarz G. Estimating the dimension of a model. The annals of statistics. 1978;6(1978):461-4.

[4] Westfall PH, Young SS. Resampling-based multiple testing: Examples and methods for p-value adjustment: John Wiley & Sons, 1993.

[5] Gormley P, Anttila V, Winsvold BS, Palta P, Esko T, Pers TH, et al. Meta-analysis of 375,000 individuals identifies 38 susceptibility loci for migraine. Nat Genet. 2016;48(2016):856-66.

[6] Schurks M, Buring JE, Kurth T. Agreement of self-reported migraine with ICHD-II criteria in the Women's Health Study. Cephalalgia : an international journal of headache. 2009;29(2009):1086-90.

[7] Linzer DA, Lewis JB. poLCA: An R package for polytomous variable latent class analysis. Journal of Statistical Software. 2011;42(2011):1-29.

[8] Vrieze SI. Model selection and psychological theory: a discussion of the differences between the Akaike information criterion (AIC) and the Bayesian information criterion (BIC). Psychol Methods. 2012;17(2012):228-43.

Supplementary Table 1: Description and ICHD-2 diagnostic criteria of migraine without aura and migraine with aura

1.1 Migraine without aura

Description: Recurrent headache disorder manifesting in attacks p lasting 4–72 hours. Typical characteristics of the headache are unilateral location, pulsating quality, moderate or severe intensity, aggravation by routine physical activity and association with nausea and/or photophobia and phonophobia.

Diagnostic criteria:

A. At least 5 attacks1fulfilling criteria B–D.
B. Headache attacks lasting 4–72 hours (untreated or unsuccessfully treated)
C. Headache has at least two of the following characteristics: 1. unilateral location, 2. pulsating quality, 3. moderate or severe pain intensity, 4. aggravation by or causing avoidance of routine physical activity (e.g. walking or climbing stairs)
D. During headache at least one of the following: 1. nausea and/or vomiting, 2. photophobia and phonophobia
E. Not attributed to another disorder

1.2.1 Typical aura with migraine headache

Description: Typical aura consisting of visual and/or sensory and/or speech symptoms. Gradual development, duration no longer than one hour, a mix of positive and negative features and complete reversibility characterize the aura which is associated with a headache fulfilling criteria for 1.1 Migraine without aura.

Diagnostic criteria:

A. At least 2 attacks fulfilling criteria B–D
B. Aura consisting of at least one of the following, but no motor weakness: 1. fully reversible visual symptoms including positive features (eg, flickering lights, spots or lines) and/or negative features (ie, loss of vision), 2. fully reversible sensory symptoms including positive features (ie, pins and needles) and/or negative features (ie, numbness), 3. fully reversible dysphasic speech disturbance
C. At least two of the following: 1. homonymous visual symptoms1and/or unilateral sensory symptoms, 2. at least one aura symptom develops gradually over ≥5 minutes and/or different aura symptoms occur in succession over ≥5 minutes, 3. each symptom lasts ≥5 and ≤60 minutes
D. Headache fulfilling criteria B–D for 1.1 Migraine without aura begins during the aura or follows aura within 60 minutes
E. Not attributed to another disorder

1.6 Probable migraine

Description: Attacks and/or headache missing one of the features needed to fulfill all criteria for a disorder coded above

Supplementary Table 2: Summary of the 46 candidate SNPs in the WGHS

Locus	SNP	chr:position	EA	AA	GWAS Priority	EAF
PRDM16	rs10218452	1:3159033	A	G	1	0.768
PRDM16	rs12135062	1:3186748	G	T	2	0.764
1p31.1	rs1572668	1:73434059	G	A	1	0.478
Near TSPAN2-NGF	rs2078371	1:115134562	T	C	1	0.883
Near TSPAN2-NGF	rs7544256	1:115281777	A	G	2	0.629
Near ADAMTSL4-ECMI	rs6693567	1:150538184	T	C	1	0.742
MEF2D	rs1925950	1:156480948	A	G	1	0.664
CARF	rs138556413	2:202968144	C	T	1	0.966
TRPM8-HJURP	rs566529	2:233848165	G	T	2	0.847
TRPM8-HJURP	rs6724624	2:233911933	C	G	T	0.806
TRPM8-HJURP	rs10166942	2:233916448	T	C	1	0.806
Near GPR149	rs13078967	3:154572157	A	C	1	0.974
Near TGFBR2	rs6791480	3:30439067	C	T	1	0.692
Near REST-SPINK2	rs7684253	4:56861145	C	T	1	0.456
Near NOTCH4	rs140002913	6:32238272	G	A	1	0.918
KCNK5	rs10456100	6:39215694	C	T	1	0.725
Near GJA1	rs28455731	6:121524892	G	T	1	0.845
HEY2-NCOA7	rs1268083	6:125727894	T	C	1	0.536
PHACTR1	rs9349379	6:12903725	A	G	1	0.564
FHL5-UFL1	rs4839827	6:96406091	C	T	2	0.476
FHL5-UFL1	rs67338227	6:96594271	A	T	1	0.769
FHL5-UFL1	rs7775721	6:96609103	C	T	3	0.662
C7orf10	rs186166891	7:40367277	A	T	1	0.900
Near DOCK4-IMMP2L	rs10155855	7:111688341	A	T	1	0.938
ASTN2	rs6478241	9:116490350	G	A	1	0.638
NRP1	rs2506142	10:33179196	A	G	1	0.819
PLCE1	rs10786156	10:94254865	C	G	1	0.556
PLCE1	rs75473620	10:94259272	A	T	2	0.962
HPSE2	rs12260159	10:98942980	G	A	1	0.923
ARMS2-HTRA1	rs2223089	10:122450644	G	C	1	0.913
MRVI1	rs4910165	11:10652497	G	C	1	0.683
MPPED2	rs11031122	11:30525891	T	C	1	0.754
YAP1	rs10895275	11:102212877	T	A	1	0.662
IGSF9B	rs561561	11:133959811	A	T	1	0.886
Near FGF6	rs1024905	12:4408974	G	C	1	0.465
LRP1	rs11172055	12:56914476	A	T	2	0.484
LRP1	rs11172113	12:57133500	T	C	1	0.590
Near ITPK1	rs11624776	14:93129246	A	C	1	0.682
CFDP1	rs77505915	16:75408245	T	G	1	0.456
Near ZCCHC14	rs4081947	16:87546264	A	G	1	0.664
Near WSCD1-NLRP1	rs75213074	17:5709320	C	T	1	0.963

Supplementary Table 2 (continued):

Locus	SNP	chr:position	EA	AA	GWAS Priority	EAF
RNF213	rs17857135	17:80288362	T	C	1	0.829
Near JAG1	rs111404218	20:10703511	C	G	1	0.670
SLC24A3	rs4814864	20:19489173	G	C	1	0.740
Near CCM2L-HCK	rs144017103	20:32041179	C	T	1	0.975
Near MED14-USP9X	rs12845494	X:40905504	C	G	1	0.723

EA = Encoded allele
AA = Alternative allele
EAF = Frequency of encoded allele

Supplementary Table 3: Corrected p-values of the statistical model selection procedure with the BIC for aura and migraine characteristics in the three nested migraineur samples after correction by permutations for testing across all characteristics

			Migraine Characteristic									
**Locus**	**CHR:Pos**	**SNP**	**Aura**	**Pulsation**	**Unipain**	**Sound**	**Light**	**Longdur**	**Nausea**	**AggrPhs**	**Inhibit**	**Freq**
PRDM16	1:3159033	**rs10218452**	-	-	-	-	0.008	-	0.003	-	0.013	0.009
			0.001	0.001	<0.001	0.001	<0.001	<0.001	<0.001	0.001	<0.001	0.001
			-	-	-	0.009	0.004	-	<0.001	0.005	0.001	-
PRDM16	1:3186748	**rs12135062**	-	-	-	-	-	-	-	-	-	-
			-	-	-	-	-	-	-	-	0.007	0.017
			-	-	-	-	-	-	-	-	0.017	-
near TSPAN2-NGF	1:115134562	**rs2078371**	<0.001	<0.001	<0.001	<0.001	<0.001	<0.001	<0.001	<0.001	<0.001	<0.001
			0.002	0.002	0.001	0.002	<0.001	0.001	0.001	<0.001	0.002	<0.001
			0.001	<0.001	<0.001	0.001	<0.001	<0.001	<0.001	<0.001	0.001	<0.001
TRPM8	2:233911933	**rs6724624**	-	-	-	-	-	-	-	-	-	-
			0.001	0.001	0.002	<0.001	<0.001	0.002	<0.001	0.001	<0.001	0.001
			<0.001	<0.001	<0.001	<0.001	<0.001	<0.001	<0.001	<0.001	<0.001	<0.001
TRPM8	2:233916448	**rs10166942**	-	-	-	-	-	-	-	-	-	-
			0.001	0.001	0.002	<0.001	<0.001	0.002	<0.001	0.001	<0.001	0.001
			<0.001	<0.001	<0.001	<0.001	<0.001	<0.001	<0.001	<0.001	<0.001	<0.001
Near TGFBR2	3:30439067	**rs6791480**	-	-	-	-	-	-	-	-	0.013	-
			-	-	-	-	-	-	-	-	-	-
			-	-	-	-	-	-	-	-	-	-

Supplementary Table 3 (continued):

			Migraine Characteristic									
**Locus**	**CHR:Pos**	**SNP**	**Aura**	**Pulsation**	**Unipain**	**Sound**	**Light**	**Longdur**	**Nausea**	**AggrPhs**	**Inhibit**	**Freq**
			-	-	-	-	-	-	-	0.002	-	-
FHL5-UFL1	6:96594271	**rs67338227**	-	-	-	-	-	-	-	-	-	-
			-	-	-	-	-	-	-	0.020	-	-
			-	-	-	-	-	-	-	0.009	-	-
FHL5-UFL1	6:96609103	**rs7775721**	0.008	0.006	0.014	0.004	0.019	0.017	0.010	0.018	0.015	0.012
			0.023	-	0.005	-	-	0.016	0.011	0.014	-	-
			0.002	-	-	-	-	-	-	-	-	-
HPSE2	10:98942980	**rs12260159**	-	-	-	-	-	-	-	-	-	-
			-	-	-	-	-	-	-	-	-	-
			-	-	-	-	-	-	-	-	-	-
MRVI1	11:10652497	**rs4910165**	-	-	-	-	-	-	0.006	-	-	-
			-	-	-	-	-	-	-	-	-	-
			-	-	-	-	-	-	-	-	-	-
IGSF9B	11:133959811	**rs561561**	-	-	-	0.008	-	-	0.001	-	-	-
			-	-	-	-	-	-	-	-	-	-
			-	-	-	-	-	-	-	-	-	-
MPPED2	11:30525891	**rs11031122**	-	-	-	-	-	-	-	-	-	-
			0.015	-	-	-	-	-	-	-	-	-
			-	-	-	-	-	-	-	-	-	-
Near FGF6	12:4408974	**rs1024905**	0.014	-	-	-	-	-	-	-	-	-
			-	-	-	-	-	-	-	-	-	-

Supplementary Table 3 (continued):

Locus	CHR:Pos	SNP	Migraine Characteristic									
			**Aura**	**Pulsation**	**Unipain**	**Sound**	**Light**	**Longdur**	**Nausea**	**AggrPhs**	**Inhibit**	**Freq**
LRP1	12:57133500	**rs11172113**	-	-	-	0.009	-	-	-	-	-	-
			0.001	0.001	0.001	<0.001	0.001	<0.001	0.001	0.001	0.001	<0.001
			0.003	0.004	<0.001	0.003	0.003	0.002	0.004	0.004	0.002	0.002
RNF213	17:80288362	**rs17857135**	0.007	0.012	0.004	0.003	0.009	0.012	0.012	0.010	0.009	0.006
			0.002	0.001	0.002	0.002	0.003	0.002	0.001	0.001	0.002	0.002
			-	-	0.011	0.003	-	0.004	0.003	-	-	-

Migraine characteristics: aura, pulsation, unipain (= unilateral pain), sound (= phonophobia), light (= photophobia), longdur (= duration of 4–72 hours), nausea, aggrphys (= aggravation by physical activity), inhibit (= severity inhibits daily activities), freq (= ≥6 attacks/year).

P-value corrections are based on permutations for testing across all characteristics (see Methods).

Lightly shaded rows represent the full migraineurs, moderately shaded areas represent the full and probable migraineurs, and heavily shaded rows represent all migraineurs (see Methods).

Supplementary Table 4: Odds-ratios (95% CI)^ for association with migraine characterized by presence/absence of aura or the diagnostic features at BIC selected SNPs

chr:pos	candidate gene	SNP	A1/A2[@]	mig. group	active migraine	aura	pulsating pain	unilateral pain	phonphobia	photophobia
1:3159033	*PRDM16*	rs10218452	A/G	full mig.	0.85 (0.78-0.93)**	0.84 (0.73-0.97)*/0.86 (0.77-0.97)*	0.87 (0.78-0.97)*/0.81 (0.68-0.97)*	0.88 (0.79-0.98)*/0.78 (0.65-0.93)*	0.82 (0.73-0.92)**/0.91 (0.78-1.05)	0.83 (0.75-0.92)**/0.99 (0.80-1.23)
				full & prob. mig.	0.85 (0.79-0.92)**	0.84 (0.75-0.94)*/0.86 (0.79-0.95)*	0.86 (0.79-0.95)*/0.84 (0.74-0.94)*	0.88 (0.81-0.96)*/0.80 (0.70-0.91)**	0.82 (0.74-0.91)**/0.89 (0.80-0.99)*	0.83 (0.76-0.91)**/0.93 (0.80-1.07)
				all migraine	0.90 (0.84-0.96)*	0.92 (0.83-1.01)/0.89 (0.82-0.97)*	0.90 (0.82-0.98)*/0.91 (0.83-1.00)*	0.91 (0.83-0.98)*/0.89 (0.81-0.99)*	0.83 (0.75-0.92)**/0.95 (0.87-1.04)	0.86 (0.79-0.93)**/1.00 (0.89-1.11)
1:3186748	*PRDM16*	rs12135062	G/T	full mig.	0.89 (0.81-0.97)*	0.89 (0.77-1.02)/0.89 (0.80-1.00)*	0.87 (0.79-0.96)*/0.97 (0.81-1.16)	0.92 (0.83-1.02)/0.80 (0.67-0.96)*	0.89 (0.80-0.99)*/0.90 (0.78-1.03)	0.87 (0.79-0.96)*/1.00 (0.81-1.23)
				full & prob. mig.	0.89 (0.83-0.95)*	0.91 (0.81-1.02)/0.88 (0.80-0.96)*	0.87 (0.80-0.95)*/0.92 (0.82-1.04)	0.90 (0.82-0.97)*/0.88 (0.78-0.99)*	0.91 (0.83-1.01)/0.87 (0.78-0.96)*	0.88 (0.81-0.96)*/0.91 (0.79-1.04)
				all migraine	0.91 (0.86-0.97)*	0.95 (0.87-1.05)/0.89 (0.82-0.97)*	0.88 (0.81-0.95)*/0.96 (0.88-1.05)	0.91 (0.84-0.99)*/0.92 (0.84-1.02)	0.90 (0.82-0.99)*/0.93 (0.86-1.00)	0.89 (0.83-0.96)*/0.96 (0.86-1.07)
1:115134562	*near TSPAN2-NGF*	rs2078371	T/C	full mig.	0.77 (0.69-0.86)**	0.77 (0.65-0.91)*/0.77 (0.67-0.89)**	0.76 (0.67-0.86)**/0.82 (0.66-1.02)	0.74 (0.66-0.84)**/0.87 (0.69-1.10)	0.74 (0.65-0.85)**/0.83 (0.69-0.99)*	0.76 (0.67-0.86)**/0.82 (0.64-1.06)
				full & prob. mig.	0.83 (0.76-0.91)**	0.87 (0.75-1.01)/0.81 (0.73-0.91)**	0.82 (0.74-0.92)**/0.85 (0.73-0.98)*	0.80 (0.71-0.89)**/0.93 (0.79-1.09)	0.78 (0.69-0.88)**/0.90 (0.79-1.03)	0.82 (0.74-0.92)**/0.86 (0.72-1.02)
				all migraine	0.85 (0.78-0.92)**	0.87 (0.77-0.99)*/0.83 (0.75-0.91)**	0.82 (0.74-0.92)**/0.87 (0.77-0.97)*	0.80 (0.72-0.89)**/0.92 (0.81-1.04)	0.77 (0.69-0.87)**/0.90 (0.81-1.00)	0.82 (0.74-0.90)**/0.90 (0.79-1.03)
2:233911933	*TRPM8*	rs6724624	C/G	full mig.	1.14 (1.03-1.26)*	1.09 (0.93-1.27)/1.18 (1.04-1.35)*	1.11 (0.99-1.25)/1.26 (1.03-1.54)*	1.15 (1.03-1.29)*/1.12 (0.91-1.37)	1.09 (0.96-1.23)/1.24 (1.06-1.46)*	1.15 (1.03-1.28)*/1.14 (0.91-1.43)
				full & prob. mig.	1.18 (1.09-1.28)**	1.14 (1.00-1.29)/1.21 (1.09-1.34)**	1.15 (1.04-1.27)*/1.24 (1.08-1.41)*	1.18 (1.07-1.30)**/1.18 (1.02-1.35)*	1.11 (0.99-1.23)/1.27 (1.13-1.42)**	1.17 (1.06-1.28)*/1.22 (1.04-1.42)*
				all migraine	1.17 (1.09-1.26)**	1.12 (1.00-1.24)*/1.20 (1.10-1.32)**	1.16 (1.05-1.28)*/1.18 (1.06-1.30)*	1.19 (1.09-1.30)**/1.14 (1.02-1.27)*	1.13 (1.01-1.26)*/1.20 (1.09-1.31)**	1.16 (1.07-1.27)**/1.18 (1.05-1.33)*
2:233916448	*TRPM8*	rs10166942	T/C	full mig.	1.14 (1.03-1.26)*	1.08 (0.93-1.27)/1.18 (1.04-1.34)*	1.11 (0.99-1.24)/1.26 (1.03-1.54)*	1.15 (1.03-1.29)*/1.12 (0.91-1.37)	1.09 (0.96-1.23)/1.24 (1.06-1.46)*	1.14 (1.02-1.28)*/1.14 (0.91-1.43)
				full & prob. mig.	1.18 (1.09-1.28)**	1.14 (1.00-1.29)/1.21 (1.09-1.33)**	1.15 (1.04-1.27)*/1.24 (1.08-1.41)*	1.18 (1.07-1.30)**/1.18 (1.02-1.35)*	1.11 (0.99-1.23)/1.27 (1.13-1.42)**	1.17 (1.06-1.28)*/1.22 (1.04-1.42)*
				all migraine	1.17 (1.09-1.25)**	1.12 (1.00-1.24)*/1.20 (1.10-1.32)**	1.16 (1.05-1.27)*/1.18 (1.06-1.30)*	1.19 (1.08-1.30)**/1.14 (1.02-1.27)*	1.13 (1.01-1.25)*/1.20 (1.09-1.31)**	1.16 (1.06-1.26)**/1.18 (1.05-1.33)*
3:30439067	*Near TGFBR2*	rs6791480	C/T	full mig.	0.89 (0.82-0.97)*	0.90 (0.79-1.03)/0.88 (0.80-0.98)*	0.90 (0.82-0.99)*/0.86 (0.73-1.01)	0.89 (0.81-0.98)*/0.89 (0.75-1.05)	0.89 (0.80-0.99)*/0.89 (0.78-1.01)	0.87 (0.80-0.95)*/0.99 (0.82-1.19)
				full & prob. mig.	0.93 (0.87-1.00)*	0.98 (0.88-1.09)/0.91 (0.83-0.98)*	0.93 (0.86-1.01)/0.93 (0.84-1.04)	0.93 (0.86-1.01)/0.94 (0.83-1.05)	0.89 (0.82-0.98)*/0.97 (0.89-1.07)	0.91 (0.84-0.98)*/1.01 (0.89-1.15)
				all migraine	0.97 (0.91-1.03)	0.98 (0.90-1.08)/0.96 (0.89-1.03)	0.96 (0.89-1.04)/0.97 (0.89-1.06)	0.94 (0.88-1.02)/1.00 (0.92-1.10)	0.90 (0.82-0.98)*/1.02 (0.95-1.10)	0.92 (0.86-0.99)*/1.07 (0.97-1.18)
6:96594271	*FHL5-UFL1*	rs67338227	A/T	full mig.	0.87 (0.79-0.95)*	0.84 (0.73-0.96)*/0.89 (0.80-1.00)	0.88 (0.80-0.98)*/0.83 (0.70-1.00)*	0.88 (0.79-0.98)*/0.84 (0.70-1.01)	0.87 (0.77-0.97)*/0.88 (0.76-1.01)	0.86 (0.78-0.95)*/0.90 (0.73-1.11)
				full & prob. mig.	0.89 (0.82-0.95)*	0.91 (0.81-1.02)/0.88 (0.80-0.96)*	0.89 (0.81-0.98)*/0.88 (0.78-0.99)*	0.88 (0.81-0.96)*/0.90 (0.79-1.02)	0.87 (0.79-0.96)*/0.91 (0.82-1.01)	0.89 (0.81-0.96)*/0.90 (0.78-1.04)
				all migraine	0.91 (0.85-0.97)*	0.95 (0.86-1.05)/0.89 (0.82-0.97)*	0.90 (0.82-0.98)*/0.93 (0.85-1.03)	0.89 (0.82-0.96)*/0.96 (0.87-1.06)	0.88 (0.80-0.97)*/0.94 (0.87-1.03)	0.91 (0.84-0.99)*/0.92 (0.83-1.03)
6:96609103	*FHL5-UFL1*	rs7775721	C/T	full mig.	0.88 (0.81-0.95)*	0.86 (0.76-0.98)*/0.89 (0.80-0.98)*	0.88 (0.80-0.96)*/0.87 (0.74-1.01)	0.87 (0.80-0.95)*/0.89 (0.76-1.05)	0.88 (0.80-0.97)*/0.87 (0.77-0.99)*	0.87 (0.80-0.95)*/0.91 (0.76-1.10)
				full & prob. mig.	0.89 (0.84-0.95)**	0.93 (0.83-1.03)/0.88 (0.81-0.95)*	0.90 (0.83-0.97)*/0.89 (0.80-0.99)*	0.87 (0.81-0.94)**/0.94 (0.84-1.05)	0.88 (0.80-0.96)*/0.91 (0.83-1.00)*	0.88 (0.82-0.95)**/0.93 (0.82-1.05)
				all migraine	0.92 (0.86-0.97)*	0.97 (0.89-1.06)/0.89 (0.83-0.95)*	0.89 (0.82-0.96)*/0.95 (0.88-1.03)	0.89 (0.83-0.95)*/0.97 (0.89-1.06)	0.88 (0.81-0.96)*/0.95 (0.88-1.02)	0.91 (0.85-0.98)*/0.93 (0.85-1.02)
10:98942980	*HPSE2*	rs12260159	G/A	full mig.	1.22 (1.03-1.44)*	0.93 (0.74-1.18)/1.51 (1.20-1.89)**	1.18 (0.98-1.42)/1.40 (0.99-1.97)	1.16 (0.96-1.39)/1.52 (1.05-2.20)*	1.15 (0.94-1.42)/1.35 (1.03-1.77)*	1.29 (1.07-1.55)*/1.00 (0.71-1.42)
				full & prob. mig.	1.14 (1.00-1.30)*	0.99 (0.82-1.21)/1.26 (1.07-1.49)*	1.16 (0.98-1.36)/1.14 (0.93-1.40)	1.11 (0.95-1.29)/1.26 (1.00-1.58)	1.18 (0.98-1.41)/1.13 (0.94-1.35)	1.17 (1.01-1.36)*/1.09 (0.86-1.39)
				all migraine	1.12 (1.00-1.25)	1.03 (0.87-1.22)/1.20 (1.04-1.39)*	1.16 (0.99-1.35)/1.10 (0.94-1.28)	1.12 (0.97-1.29)/1.14 (0.96-1.36)	1.14 (0.96-1.36)/1.12 (0.97-1.29)	1.14 (1.00-1.31)/1.10 (0.92-1.33)
11:10652497	*MRVI1*	rs4910165	G/C	full mig.	1.10 (1.01-1.20)*	1.21 (1.05-1.38)*/1.04 (0.94-1.15)	1.07 (0.97-1.18)/1.20 (1.01-1.42)*	1.08 (0.98-1.19)/1.17 (0.98-1.39)	1.11 (1.00-1.23)/1.09 (0.95-1.24)	1.14 (1.04-1.25)*/0.95 (0.79-1.14)
				full & prob. mig.	1.08 (1.01-1.15)*	1.15 (1.03-1.28)*/1.04 (0.95-1.13)	1.03 (0.95-1.12)/1.17 (1.04-1.30)*	1.06 (0.98-1.15)/1.10 (0.98-1.24)	1.08 (0.98-1.18)/1.07 (0.97-1.18)	1.11 (1.02-1.20)*/0.99 (0.88-1.13)
				all migraine	1.04 (0.98-1.10)	1.09 (1.00-1.20)/1.01 (0.93-1.08)	1.03 (0.95-1.11)/1.06 (0.97-1.15)	1.05 (0.97-1.13)/1.02 (0.94-1.12)	1.09 (1.00-1.19)/1.01 (0.93-1.08)	1.08 (1.01-1.16)*/0.96 (0.88-1.06)
11:30525891	*MPPED2*	rs11031122	T/C	full mig.	0.94 (0.86-1.03)	0.84 (0.74-0.97)*/1.02 (0.91-1.14)	0.95 (0.86-1.05)/0.92 (0.77-1.09)	0.95 (0.86-1.04)/0.94 (0.78-1.12)	0.94 (0.85-1.06)/0.94 (0.82-1.08)	0.95 (0.86-1.04)/0.94 (0.77-1.14)
				full & prob. mig.	0.95 (0.89-1.02)	0.85 (0.76-0.95)*/1.02 (0.94-1.12)	0.97 (0.88-1.05)/0.94 (0.84-1.05)	0.98 (0.90-1.07)/0.90 (0.80-1.01)	0.93 (0.85-1.03)/0.98 (0.89-1.09)	0.94 (0.87-1.02)/1.00 (0.88-1.15)
				all migraine	0.95 (0.89-1.01)	0.86 (0.78-0.94)*/1.02 (0.94-1.11)	1.00 (0.92-1.09)/0.90 (0.83-0.99)*	0.99 (0.92-1.07)/0.90 (0.82-0.99)*	0.94 (0.86-1.03)/0.96 (0.89-1.04)	0.93 (0.86-1.00)*/1.00 (0.90-1.11)
11:133959811	*IGSF9B*	rs561561	A/T	full mig.	1.16 (1.02-1.32)*	1.16 (0.94-1.42)/1.17 (0.99-1.37)	1.18 (1.01-1.36)*/1.12 (0.87-1.44)	1.21 (1.04-1.40)*/1.02 (0.79-1.31)	1.06 (0.90-1.24)/1.37 (1.11-1.70)*	1.15 (1.00-1.33)*/1.20 (0.89-1.62)
				full & prob. mig.	1.16 (1.05-1.29)*	1.15 (0.98-1.36)/1.16 (1.02-1.33)*	1.17 (1.03-1.33)*/1.14 (0.96-1.35)	1.15 (1.02-1.31)*/1.17 (0.98-1.40)	1.06 (0.92-1.21)/1.29 (1.11-1.51)**	1.15 (1.02-1.30)*/1.19 (0.98-1.45)
				all migraine	1.09 (0.99-1.19)	1.08 (0.94-1.24)/1.08 (0.97-1.21)	1.12 (1.00-1.27)/1.04 (0.92-1.18)	1.13 (1.00-1.26)*/1.03 (0.90-1.17)	1.03 (0.90-1.17)/1.13 (1.00-1.27)*	1.10 (0.99-1.23)/1.05 (0.91-1.22)
12:4408974	*Near FGF6*	rs1024905	G/C	full mig.	1.10 (1.02-1.19)*	1.03 (0.91-1.16)/1.15 (1.04-1.27)*	1.11 (1.02-1.22)*/1.06 (0.91-1.24)	1.11 (1.02-1.22)*/1.06 (0.90-1.24)	1.12 (1.02-1.24)*/1.07 (0.95-1.21)	1.12 (1.03-1.22)*/1.03 (0.86-1.23)
				full & prob. mig.	1.08 (1.01-1.15)*	0.97 (0.88-1.08)/1.15 (1.06-1.24)**	1.08 (1.00-1.17)/1.08 (0.98-1.20)	1.10 (1.02-1.19)*/1.04 (0.94-1.16)	1.09 (1.00-1.19)*/1.07 (0.98-1.17)	1.09 (1.02-1.18)*/1.04 (0.93-1.18)
				all migraine	1.05 (0.99-1.11)	0.95 (0.88-1.04)/1.12 (1.05-1.20)*	1.08 (1.00-1.16)*/1.03 (0.95-1.11)	1.10 (1.02-1.17)*/0.99 (0.91-1.08)	1.07 (0.99-1.17)/1.04 (0.97-1.12)	1.07 (1.00-1.14)/1.03 (0.94-1.12)
12:57133500	*LRP1*	rs11172113	T/C	full mig.	1.12 (1.03-1.21)*	1.03 (0.91-1.17)/1.17 (1.06-1.30)*	1.13 (1.03-1.23)*/1.09 (0.93-1.27)	1.13 (1.03-1.23)*/1.09 (0.92-1.27)	1.04 (0.94-1.15)/1.25 (1.10-1.42)**	1.11 (1.02-1.22)*/1.12 (0.94-1.34)
				full & prob. mig.	1.14 (1.07-1.22)**	1.10 (0.99-1.22)/1.16 (1.07-1.25)**	1.13 (1.04-1.22)*/1.15 (1.04-1.28)*	1.12 (1.04-1.21)*/1.17 (1.05-1.31)*	1.09 (1.00-1.18)/1.19 (1.09-1.31)**	1.13 (1.05-1.21)*/1.16 (1.03-1.31)*
				all migraine	1.12 (1.06-1.18)**	1.06 (0.97-1.16)/1.15 (1.07-1.24)**	1.12 (1.04-1.21)*/1.11 (1.02-1.20)*	1.11 (1.04-1.19)*/1.12 (1.03-1.22)*	1.07 (0.99-1.17)/1.14 (1.06-1.23)**	1.10 (1.03-1.18)*/1.14 (1.04-1.25)*
17:80288362	*RNF213*	rs17857135	T/C	full mig.	0.85 (0.77-0.94)**	0.81 (0.70-0.94)*/0.88 (0.78-0.99)*	0.85 (0.76-0.95)*/0.84 (0.70-1.02)	0.86 (0.77-0.96)*/0.83 (0.68-1.00)	0.83 (0.73-0.93)*/0.90 (0.77-1.04)	0.86 (0.77-0.96)*/0.82 (0.66-1.02)
				full & prob. mig.	0.86 (0.79-0.93)**	0.86 (0.76-0.98)*/0.86 (0.78-0.95)*	0.87 (0.79-0.96)*/0.84 (0.74-0.96)*	0.87 (0.79-0.95)*/0.84 (0.74-0.96)*	0.82 (0.74-0.91)**/0.90 (0.81-1.01)	0.87 (0.79-0.95)*/0.84 (0.72-0.97)*
				all migraine	0.90 (0.84-0.96)*	0.91 (0.82-1.02)/0.89 (0.81-0.97)*	0.89 (0.81-0.98)*/0.90 (0.81-0.99)*	0.86 (0.79-0.94)**/0.95 (0.85-1.06)	0.82 (0.74-0.91)**/0.95 (0.87-1.04)	0.90 (0.83-0.98)*/0.88 (0.78-0.99)*

^Odds-ratios and 95% CI from logistic regression adjusted for 10 principal components of sub-European population substructure. [@]A1/A2 encoded allele/reference allele

Legend

BIC selected model	logistic regression p-value	
basic model	*	$p < 0.05$
subset model	**	$p < 0.001$
inverse subset model		

Supplementary Table 4: (continued)

chr:pos	candidate gene	SNP	A1/A2[@]	mig. group	active migraine	miglongdur.OR	mignausea.OR	migaggrphys.OR	miginhibit.OR	migfreq.OR
1:3159033	*PRDM16*	rs10218452	A/G	full mig.	0.85 (0.78-0.93)**	0.85 (0.78-0.94)**/1.00 (0.00-Inf)	0.82 (0.75-0.91)**/1.29 (0.93-1.80)	0.82 (0.72-0.92)**/0.90 (0.79-1.04)	0.83 (0.75-0.92)**/0.94 (0.77-1.13)	0.93 (0.80-1.07)/0.81 (0.72-0.91)**
				full & prob. mig.	0.85 (0.79-0.92)**	0.87 (0.80-0.94)**/0.69 (0.52-0.92)*	0.83 (0.77-0.90)**/0.96 (0.81-1.13)	0.80 (0.72-0.89)**/0.90 (0.81-0.99)*	0.80 (0.73-0.88)**/0.96 (0.85-1.08)	0.91 (0.81-1.03)/0.82 (0.75-0.90)**
				all migraine	0.90 (0.84-0.96)*	0.89 (0.83-0.96)*/0.93 (0.82-1.07)	0.84 (0.77-0.91)**/1.04 (0.93-1.16)	0.81 (0.73-0.90)**/0.95 (0.88-1.03)	0.82 (0.75-0.90)**/0.99 (0.90-1.09)	0.95 (0.85-1.06)/0.88 (0.81-0.95)*
1:3186748	*PRDM16*	rs12135062	G/T	full mig.	0.89 (0.81-0.97)*	0.89 (0.82-0.97)*/1.00 (0.00-Inf)	0.90 (0.82-0.98)*/0.85 (0.65-1.13)	0.85 (0.76-0.96)*/0.94 (0.83-1.08)	0.87 (0.79-0.96)*/0.97 (0.81-1.16)	0.96 (0.84-1.10)/0.85 (0.76-0.95)*
				full & prob. mig.	0.89 (0.83-0.95)*	0.90 (0.84-0.97)*/0.72 (0.55-0.94)*	0.89 (0.82-0.96)*/0.90 (0.77-1.05)	0.86 (0.78-0.96)*/0.91 (0.83-1.00)	0.85 (0.77-0.92)**/0.97 (0.87-1.09)	0.95 (0.85-1.07)/0.85 (0.78-0.93)**
				all migraine	0.91 (0.86-0.97)*	0.92 (0.86-0.99)*/0.89 (0.78-1.01)	0.90 (0.83-0.97)*/0.95 (0.86-1.06)	0.86 (0.78-0.95)*/0.95 (0.88-1.02)	0.86 (0.79-0.93)**/0.98 (0.90-1.07)	0.97 (0.87-1.07)/0.89 (0.82-0.96)*
1:115134562	*near TSPAN2-NGF*	rs2078371	T/C	full mig.	0.77 (0.69-0.86)**	0.77 (0.69-0.86)**/1.00 (0.00-Inf)	0.80 (0.71-0.90)**/0.54 (0.40-0.74)**	0.78 (0.67-0.90)**/0.76 (0.65-0.90)**	0.81 (0.72-0.92)*/0.65 (0.53-0.81)**	0.68 (0.58-0.80)**/0.85 (0.73-0.98)*
				full & prob. mig.	0.83 (0.76-0.91)**	0.83 (0.76-0.91)**/0.89 (0.62-1.28)	0.83 (0.75-0.92)**/0.86 (0.70-1.04)	0.81 (0.71-0.93)*/0.85 (0.75-0.96)*	0.84 (0.75-0.94)*/0.82 (0.71-0.95)*	0.72 (0.63-0.82)**/0.93 (0.82-1.04)
				all migraine	0.85 (0.78-0.92)**	0.84 (0.77-0.92)**/0.86 (0.73-1.01)	0.84 (0.76-0.92)**/0.86 (0.76-0.98)*	0.82 (0.72-0.93)*/0.86 (0.78-0.95)*	0.85 (0.76-0.95)*/0.84 (0.75-0.94)*	0.73 (0.65-0.83)**/0.92 (0.83-1.02)
2:233911933	*TRPM8*	rs6724624	C/G	full mig.	1.14 (1.03-1.26)*	1.15 (1.04-1.27)*/1.00 (0.00-Inf)	1.17 (1.05-1.30)*/0.94 (0.69-1.27)	1.12 (0.99-1.28)/1.17 (1.01-1.36)*	1.12 (1.00-1.26)*/1.22 (0.99-1.50)	1.23 (1.05-1.44)*/1.09 (0.96-1.24)
				full & prob. mig.	1.18 (1.09-1.28)**	1.16 (1.06-1.26)**/1.69 (1.18-2.43)*	1.22 (1.11-1.33)**/1.05 (0.89-1.25)	1.15 (1.02-1.30)*/1.20 (1.08-1.34)**	1.17 (1.06-1.30)*/1.19 (1.05-1.36)*	1.23 (1.08-1.39)*/1.15 (1.04-1.27)*
				all migraine	1.17 (1.09-1.26)**	1.14 (1.05-1.23)*/1.28 (1.10-1.48)*	1.22 (1.11-1.33)**/1.09 (0.97-1.22)	1.17 (1.04-1.32)*/1.16 (1.07-1.27)**	1.17 (1.06-1.29)*/1.17 (1.06-1.29)*	1.22 (1.08-1.37)*/1.14 (1.05-1.25)*
2:233916448	*TRPM8*	rs10166942	T/C	full mig.	1.14 (1.03-1.26)*	1.14 (1.03-1.26)*/1.00 (0.00-Inf)	1.17 (1.05-1.30)*/0.94 (0.69-1.27)	1.12 (0.98-1.28)/1.17 (1.01-1.36)*	1.12 (1.00-1.26)*/1.22 (0.99-1.49)	1.23 (1.05-1.44)*/1.09 (0.96-1.23)
				full & prob. mig.	1.18 (1.09-1.28)**	1.16 (1.06-1.26)**/1.68 (1.17-2.41)*	1.21 (1.11-1.33)**/1.05 (0.89-1.25)	1.15 (1.02-1.30)*/1.20 (1.08-1.33)**	1.17 (1.06-1.30)*/1.19 (1.05-1.36)*	1.23 (1.08-1.39)*/1.15 (1.04-1.27)*
				all migraine	1.17 (1.09-1.25)**	1.14 (1.05-1.23)*/1.28 (1.10-1.49)*	1.21 (1.11-1.33)**/1.09 (0.97-1.22)	1.17 (1.04-1.32)*/1.16 (1.07-1.27)**	1.17 (1.06-1.29)*/1.17 (1.06-1.29)*	1.22 (1.08-1.37)*/1.14 (1.05-1.24)*
3:30439067	*Near TGFBR2*	rs6791480	C/T	full mig.	0.89 (0.82-0.97)*	0.89 (0.82-0.97)*/1.00 (0.00-Inf)	0.88 (0.81-0.96)*/1.06 (0.81-1.39)	0.90 (0.81-1.00)/0.88 (0.78-0.99)*	0.86 (0.78-0.94)*/1.02 (0.86-1.21)	0.84 (0.75-0.96)*/0.93 (0.83-1.03)
				full & prob. mig.	0.93 (0.87-1.00)*	0.93 (0.86-0.99)*/1.02 (0.78-1.33)	0.91 (0.84-0.98)*/1.05 (0.90-1.21)	0.93 (0.85-1.03)/0.93 (0.85-1.01)	0.91 (0.84-0.99)*/0.97 (0.87-1.08)	0.89 (0.80-0.99)*/0.96 (0.88-1.04)
				all migraine	0.97 (0.91-1.03)	0.94 (0.88-1.01)/1.06 (0.94-1.20)	0.92 (0.85-0.98)*/1.08 (0.98-1.19)	0.94 (0.85-1.03)/0.98 (0.92-1.06)	0.91 (0.84-0.99)*/1.02 (0.94-1.11)	0.91 (0.83-1.00)*/1.00 (0.93-1.08)
6:96594271	*FHL5-UFL1*	rs67338227	A/T	full mig.	0.87 (0.79-0.95)*	0.87 (0.80-0.95)*/1.00 (0.00-Inf)	0.88 (0.80-0.97)*/0.81 (0.61-1.07)	0.79 (0.71-0.89)**/0.98 (0.86-1.13)	0.86 (0.77-0.95)*/0.93 (0.77-1.12)	0.84 (0.73-0.96)*/0.90 (0.80-1.01)
				full & prob. mig.	0.89 (0.82-0.95)*	0.89 (0.82-0.96)*/0.92 (0.69-1.24)	0.88 (0.81-0.96)*/0.91 (0.77-1.06)	0.84 (0.75-0.94)*/0.93 (0.84-1.02)	0.87 (0.79-0.95)*/0.93 (0.83-1.05)	0.86 (0.77-0.97)*/0.91 (0.83-0.99)*
				all migraine	0.91 (0.85-0.97)*	0.89 (0.83-0.96)*/1.01 (0.88-1.16)	0.88 (0.81-0.95)*/0.99 (0.89-1.11)	0.84 (0.75-0.93)**/0.96 (0.89-1.04)	0.87 (0.79-0.95)*/0.97 (0.89-1.06)	0.89 (0.80-0.98)*/0.93 (0.86-1.01)
6:96609103	*FHL5-UFL1*	rs7775721	C/T	full mig.	0.88 (0.81-0.95)*	0.88 (0.81-0.95)*/1.00 (0.00-Inf)	0.88 (0.81-0.96)*/0.80 (0.62-1.04)	0.83 (0.75-0.92)**/0.94 (0.84-1.06)	0.88 (0.80-0.96)*/0.88 (0.75-1.03)	0.84 (0.75-0.95)*/0.90 (0.81-1.00)*
				full & prob. mig.	0.89 (0.84-0.95)**	0.89 (0.83-0.95)**/0.92 (0.71-1.18)	0.89 (0.83-0.96)*/0.90 (0.78-1.03)	0.86 (0.78-0.94)*/0.92 (0.85-1.00)	0.89 (0.82-0.97)*/0.90 (0.81-0.99)*	0.86 (0.78-0.95)*/0.91 (0.84-0.99)*
				all migraine	0.92 (0.86-0.97)*	0.89 (0.84-0.95)**/1.01 (0.90-1.14)	0.88 (0.83-0.95)**/0.99 (0.90-1.09)	0.85 (0.78-0.94)**/0.96 (0.89-1.02)	0.90 (0.83-0.97)*/0.94 (0.87-1.01)	0.88 (0.80-0.96)*/0.94 (0.88-1.01)
10:98942980	*HPSE2*	rs12260159	G/A	full mig.	1.22 (1.03-1.44)*	1.22 (1.04-1.44)*/1.00 (0.00-Inf)	1.25 (1.05-1.48)*/1.03 (0.63-1.70)	1.26 (1.01-1.57)*/1.18 (0.93-1.51)	1.25 (1.04-1.51)*/1.14 (0.83-1.58)	1.13 (0.88-1.44)/1.30 (1.05-1.62)*
				full & prob. mig.	1.14 (1.00-1.30)*	1.16 (1.01-1.33)*/1.00 (0.62-1.61)	1.19 (1.03-1.38)*/1.02 (0.78-1.33)	1.21 (0.99-1.47)/1.11 (0.94-1.31)	1.17 (0.99-1.38)/1.12 (0.91-1.37)	1.06 (0.87-1.29)/1.22 (1.03-1.44)*
				all migraine	1.12 (1.00-1.25)	1.17 (1.03-1.33)*/0.99 (0.80-1.24)	1.18 (1.03-1.36)*/1.04 (0.87-1.24)	1.18 (0.97-1.43)/1.11 (0.96-1.27)	1.16 (0.99-1.36)/1.10 (0.94-1.28)	1.05 (0.88-1.25)/1.18 (1.02-1.35)*
11:10652497	*MRVI1*	rs4910165	G/C	full mig.	1.10 (1.01-1.20)*	1.10 (1.01-1.20)*/1.00 (0.00-Inf)	1.12 (1.02-1.22)*/0.94 (0.72-1.22)	1.19 (1.06-1.33)*/1.00 (0.88-1.12)	1.14 (1.04-1.26)*/0.97 (0.82-1.15)	1.14 (1.00-1.30)*/1.07 (0.96-1.19)
				full & prob. mig.	1.08 (1.01-1.15)*	1.08 (1.01-1.16)*/0.97 (0.75-1.26)	1.13 (1.04-1.21)*/0.90 (0.78-1.03)	1.14 (1.03-1.26)*/1.03 (0.94-1.12)	1.09 (1.01-1.19)*/1.04 (0.94-1.16)	1.14 (1.03-1.27)*/1.04 (0.95-1.13)
				all migraine	1.04 (0.98-1.10)	1.07 (1.00-1.14)/0.95 (0.84-1.07)	1.10 (1.02-1.18)*/0.94 (0.86-1.03)	1.13 (1.02-1.24)*/1.00 (0.93-1.07)	1.08 (0.99-1.17)/1.00 (0.92-1.09)	1.14 (1.03-1.25)*/0.99 (0.92-1.07)
11:30525891	*MPPED2*	rs11031122	T/C	full mig.	0.94 (0.86-1.03)	0.94 (0.86-1.03)/1.00 (0.00-Inf)	0.96 (0.87-1.05)/0.83 (0.63-1.09)	0.94 (0.83-1.05)/0.95 (0.84-1.09)	0.94 (0.85-1.04)/0.94 (0.79-1.12)	0.89 (0.78-1.02)/0.98 (0.88-1.10)
				full & prob. mig.	0.95 (0.89-1.02)	0.96 (0.89-1.03)/0.89 (0.68-1.17)	0.94 (0.86-1.01)/1.05 (0.90-1.23)	0.98 (0.88-1.09)/0.94 (0.86-1.03)	0.94 (0.86-1.03)/0.98 (0.87-1.09)	0.89 (0.80-1.00)/1.00 (0.91-1.09)
				all migraine	0.95 (0.89-1.01)	0.95 (0.89-1.02)/0.96 (0.84-1.09)	0.92 (0.86-0.99)*/1.01 (0.91-1.12)	0.98 (0.89-1.09)/0.94 (0.87-1.01)	0.94 (0.86-1.02)/0.97 (0.89-1.05)	0.93 (0.84-1.03)/0.97 (0.89-1.04)
11:133959811	*IGSF9B*	rs561561	A/T	full mig.	1.16 (1.02-1.32)*	1.16 (1.02-1.32)*/1.00 (0.00-Inf)	1.19 (1.04-1.36)*/0.93 (0.64-1.37)	1.18 (0.99-1.40)/1.14 (0.94-1.38)	1.13 (0.98-1.31)/1.28 (0.97-1.67)	1.31 (1.06-1.61)*/1.08 (0.92-1.27)
				full & prob. mig.	1.16 (1.05-1.29)*	1.17 (1.05-1.31)*/0.97 (0.66-1.43)	1.20 (1.07-1.35)*/1.01 (0.82-1.25)	1.18 (1.01-1.38)*/1.14 (1.00-1.31)	1.16 (1.01-1.31)*/1.17 (0.99-1.38)	1.23 (1.04-1.46)*/1.12 (0.98-1.27)
				all migraine	1.09 (0.99-1.19)	1.13 (1.02-1.25)*/0.95 (0.80-1.13)	1.17 (1.05-1.31)*/0.95 (0.82-1.09)	1.14 (0.98-1.32)/1.06 (0.95-1.18)	1.12 (0.99-1.27)/1.05 (0.93-1.19)	1.16 (1.00-1.35)*/1.05 (0.94-1.16)
12:4408974	*Near FGF6*	rs1024905	G/C	full mig.	1.10 (1.02-1.19)*	1.10 (1.02-1.19)*/1.00 (0.00-Inf)	1.11 (1.02-1.21)*/0.99 (0.77-1.27)	1.08 (0.98-1.20)/1.13 (1.00-1.26)*	1.09 (0.99-1.19)/1.15 (0.98-1.35)	1.17 (1.04-1.32)*/1.05 (0.95-1.17)
				full & prob. mig.	1.08 (1.01-1.15)*	1.08 (1.02-1.16)*/1.02 (0.80-1.31)	1.11 (1.04-1.19)*/0.96 (0.84-1.10)	1.07 (0.97-1.17)/1.09 (1.00-1.18)*	1.06 (0.98-1.14)/1.12 (1.02-1.24)*	1.13 (1.03-1.25)*/1.05 (0.97-1.13)
				all migraine	1.05 (0.99-1.11)	1.08 (1.01-1.15)*/0.97 (0.86-1.08)	1.10 (1.03-1.18)*/0.97 (0.88-1.06)	1.06 (0.97-1.16)/1.05 (0.98-1.12)	1.05 (0.97-1.13)/1.06 (0.98-1.14)	1.11 (1.01-1.21)*/1.03 (0.96-1.10)
12:57133500	*LRP1*	rs11172113	T/C	full mig.	1.12 (1.03-1.21)*	1.12 (1.03-1.21)*/1.00 (0.00-Inf)	1.12 (1.04-1.22)*/1.04 (0.80-1.33)	1.12 (1.01-1.25)*/1.11 (0.99-1.24)	1.10 (1.00-1.20)*/1.18 (1.00-1.38)*	1.09 (0.96-1.23)/1.14 (1.03-1.26)*
				full & prob. mig.	1.14 (1.07-1.22)**	1.14 (1.06-1.21)**/1.17 (0.91-1.50)	1.14 (1.07-1.23)**/1.11 (0.97-1.27)	1.13 (1.03-1.24)*/1.14 (1.05-1.24)*	1.13 (1.05-1.23)*/1.14 (1.03-1.27)*	1.12 (1.02-1.24)*/1.14 (1.06-1.24)**
				all migraine	1.12 (1.06-1.18)**	1.13 (1.06-1.21)**/1.05 (0.94-1.18)	1.13 (1.05-1.21)**/1.09 (0.99-1.19)	1.14 (1.04-1.25)*/1.10 (1.03-1.18)*	1.13 (1.05-1.22)*/1.10 (1.02-1.19)*	1.14 (1.04-1.25)*/1.10 (1.03-1.18)*
17:80288362	*RNF213*	rs17857135	T/C	full mig.	0.85 (0.77-0.94)**	0.85 (0.77-0.94)*/1.00 (0.00-Inf)	0.85 (0.77-0.94)*/0.86 (0.63-1.17)	0.83 (0.73-0.94)*/0.88 (0.76-1.02)	0.83 (0.75-0.93)*/0.92 (0.75-1.12)	0.79 (0.68-0.91)*/0.90 (0.79-1.02)
				full & prob. mig.	0.86 (0.79-0.93)**	0.86 (0.80-0.94)**/0.80 (0.59-1.08)	0.84 (0.77-0.91)**/0.96 (0.81-1.14)	0.84 (0.75-0.94)*/0.87 (0.79-0.97)*	0.86 (0.78-0.95)*/0.86 (0.76-0.97)*	0.80 (0.71-0.91)**/0.90 (0.81-0.99)*
				all migraine	0.90 (0.84-0.96)*	0.87 (0.80-0.94)**/1.00 (0.86-1.16)	0.85 (0.78-0.93)**/0.98 (0.88-1.11)	0.85 (0.76-0.95)*/0.92 (0.84-1.00)	0.87 (0.79-0.95)*/0.92 (0.84-1.02)	0.83 (0.75-0.93)*/0.93 (0.85-1.01)

Supplementary Table 5. Aura status and migraine characteristics of the three nested samples of the recruitment sample used in the latent class analyses

	**Full migraineurs (N=36105)**	**Full and probable migraineurs (N=54011)**	**All migraineurs (N=69,861)**
**Aura or migraine symptom**	**N (%)**	**N (%)**	**N (%)**
Aura	15284 (42.3)	21360 (39.5)	27965 (40.0)
Pulsating pain	27512 (76.2)	36016 (66.7)	39824 (57.0)
Unilateral pain	27203 (75.3)	36231 (67.1)	41218 (59.0)
Phonophobia	25145 (69.6)	32275 (59.8)	34819 (49.8)
Photophobia	31028 (85.9)	42423 (78.5)	49244 (70.5)
Duration of 4-72 hours	36105 (100.0)	50754 (94.0)	56021 (80.2)
Nausea	31945 (88.5)	42463 (78.6)	45846 (65.6)
Pain aggravation by physical activity	22115 (61.3)	26845 (49.7)	28421 (40.7)
Inhibition of daily activities	29427 (81.5)	36687 (67.9)	38971 (55.8)
